# Sustainable Synthesis of *p*-Hydroxycinnamic Diacids through Proline-Mediated Knoevenagel Condensation in Ethanol: An Access to Potent Phenolic UV Filters and Radical Scavengers

**DOI:** 10.3390/antiox9040331

**Published:** 2020-04-18

**Authors:** Benjamin Rioux, Cédric Peyrot, Matthieu M. Mention, Fanny Brunissen, Florent Allais

**Affiliations:** URD Agro-Biotechnologies Industrielles, CEBB, AgroParisTech, 51110 Pomacle, France; benjamin.rioux@agroparistech.fr (B.R.); cedric.peyrot@agroparistech.fr (C.P.); matthieu.mention@agroparistech.fr (M.M.M.); fanny.brunissen@agroparistech.fr (F.B.)

**Keywords:** Knoevenagel condensation, L-proline, *p*-hydroxycinnamic diacids, ferulic diacid, coumaric diacid, caffeic diacid, anti-UV, antioxidant

## Abstract

*p*-Hydroxycinnamic diacids are reaction intermediates of the classical Knoevenagel–Doebner condensation between malonic acid and benzaldehydes. As they are generally obtained in low yields, they remain relatively under-studied and under-exploited. Herein, we developed and optimized a sustainable synthetic procedure allowing the production of these compounds in good to high yields (60–80%) using proline as the catalyst and ethanol as the solvent. Study of their antioxidant and anti-UV activities revealed that these *p*-hydroxycinnamic diacids were not only potent radical scavengers but also efficient UV filters exhibiting high photostability.

## 1. Introduction

Faced with growing consumer demand for bio-based or natural products and the need to become less dependent on fossil resources, industry is turning to eco-friendlier products and processes. In this context, lignin and related chemicals (e.g., lignans, phenolic acids) appear as a valuable source of sustainable aromatic building blocks [1,2]. Indeed, *p*-hydroxycinnamic acids and their derivatives can be readily extracted from different biomasses and be further (chemo-)enzymatically modified [3,4,5,6] to provide them with physicochemical and biological properties of interest in various fields, such as antioxidants [7,8,9] and anti-UV compounds [10,11,12]. However, *p*-hydroxycinnamic acids and their derivatives are not only poorly water soluble, but also they are usually present in relatively small quantities in biomass, thus greatly limiting their uses at the industrial scale. To overcome the latter issue, many synthetic methods have been designed to access *p*-hydroxycinnamic acids in high yield and at large scale from commercially available reagents. Among them, the main synthetic route consists in the condensation of malonic acid with *p*-hydroxybenzaldehydes through the Knoevenagel–Doebner reaction.

Usually, Knoevenagel–Doebner reactions require a large amount of pyridine as a solvent and an amine catalyst, such as aniline or piperidine [13]. However, both reagents are toxic and induce serious health damage [14]. In order to design more sustainable synthetic procedures, few alternatives based on green chemistry principles have emerged [15]. For instance, ionic liquids can substitute pyridine and represent an interesting alternative thanks to their recyclability [16,17,18]. Nevertheless, their use is still limited in industrial scale-up [19]. Preferably, the reaction can also be carried out in water, which makes it industrially more viable [20,21,22]. When it comes to reagents, petro-sourced nitrogen catalysts can be substituted by amino acid: especially proline [23,24,25,26]. Recently Peyrot et al. described an access to naturally-occurring phenolic acids by replacing pyridine/aniline-mediated classical Knoevenagel–Doebner reaction by a proline-mediated one in ethanol while achieving excellent yields [27]. It is noteworthy to mention that the reaction time can also be shortened using a microwave-activated Knoevenagel–Doebner reaction as demonstrated by Mouterde et al. [28].

**Figure 1:**
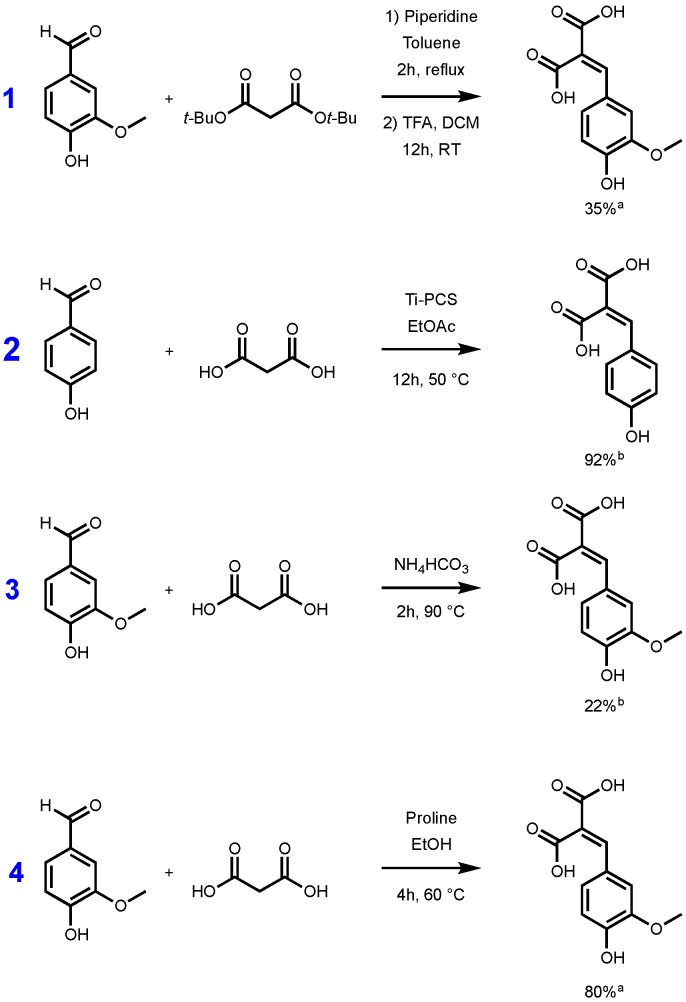
Synthetic pathways to *p*-hydroxycinnamic diacids according to Vivier et al. [31] (1), Mangala et al. [32] (2), Schijndel et al. [33] (3), and our study (4). [a] Isolated yield; [b] evaluated by HPLC.

Whatever the synthetic procedure used, Knoevenagel–Doebner condensation always produces *p*-hydroxycinnamic diacids as intermediates, and such diacids are advantageous compared to the corresponding monoacid as they are more water soluble thanks to the extra carboxylic acid moiety. Unfortunately, during the condensation, the diacids are easily transformed into the *p*-hydroxycinnamic acids through decarboxylation. In 2007, Bermudez et al. highlighted the importance of the reaction time, the base, and the temperature used for this decarboxylation step [29]. In fact, when the reaction temperature is above 50 °C, decarboxylation is favored and leads to *p*-hydroxycinnamic acids. Conversely, at low temperature, the formation of the diacid may be preferred at the expense of *p*-hydroxycinnamic acid formation. To the best of our knowledge, only a few synthetic pathways to *p*-hydroxycinnamic diacid have been reported in the literature. Conventionally, access to this type of diacid requires the protection/deprotection of the two carboxylic acid moieties functions. The use of protective group such as tert-butyl ester is well described in the literature [30,31]. Malonic acid is first esterified to provide the corresponding di-tert-butyl malonate, and the latter then undergoes a Knoevenagel reaction with the *p*-hydroxybenzaldehyde required. The reaction is generally carried out in the presence of piperidine, in toluene or ethanol [30]. The resulting diester is then deprotected in the presence of trifluoroacetic acid (TFA) generally in a modest yield (35%) [31]. Mangala et al. proposed an organometallic-based pathway using titanium polycarbosilane (Ti-PCS) in ethyl acetate, allowing them to obtain a conversion, evaluated by HPLC, higher than 90% [32]. More recently, a solvent-free approach was proposed by Schijndel et al. in the presence of ammonium bicarbonate, at 90 °C for two hours. Under these conditions, the conversion of the vanillin into the corresponding ferulic diacid was evaluated at 22% by HPLC [33]. These three synthetic pathways are highlighted in the Figure 1.

Although the reaction conditions of the above procedures are globally in line with the green chemistry principles and HPLC conversions are promising, isolated yields remain relatively low [31] or not described [32,33]. Additionally, Mangala et al.’s [32] (Figure 1, pathway 2) method uses titan derivatives (Ti-PCS) as catalyst. Titan derivatives, such as TiO_2_, are widely described as detrimental for the environment or DNA so limiting the use of such as metal catalysts would be relevant [34,35,36]. Under these considerations, in this work, we therefore sought to develop a synthetic pathway that provides *p*-hydroxycinnamic diacids in high yield and at the multigram scale while being environmentally friendly. Moreover, with *p*-hydroxycinnamic diesters being reported as having high anti-UV and antioxidant properties [10,11,37], a specific focus has also been put on the evaluation of the radical scavenging and UV filtering capacities of the *p*-hydroxycinnamic diacids.

## 2. Materials and Methods

### 2.1. Synthesis: General Procedure

Vanillin (225 mg, 1.5 mmol, 1 eq), malonic acid (153.9 mg, 1.5 mmol, 1 eq), and proline (17.1 mg, 0.15 mmol, 0.1 eq) were dissolved in ethanol (3 mL, 0.5 M) and stirred at 60 °C for four hours. The reaction mixture was then evaporated under vacuum (conversion yields were determined by ^1^H NMR) and then purified by column chromatography on a C-18 reverse silica gel (H_2_O:methanol, 80:20). A total of 282 mg of a yellow/white powder was recovered (1.18 mmol, 80%).

### 2.2. NMR Analysis

^1^H NMR spectra were achieved on a Bruker Fourier 300 (300 MHz) (Billerica, MA, USA) and calibrated with acetone-*d_6_*, with proton signals at δ 2.05 ppm. Data are reported as follows: chemical shift (δ ppm), integration, multiplicity (s = singlet, d = doublet, dd = doublet of doublets), coupling constant (Hz), and assignment. ^13^C NMR spectra were recorded on a Bruker Fourier 300 (75 MHz) (Billerica, MA, USA) and were calibrated with Acetone-*d_6_*, signals at δ = 206.26 and 29.84 ppm. Data are reported as follows: chemical shift (δ ppm) and attribution. NMR spectra assignments were achieved using COSY, HMBC, and HSQC spectra.

### 2.3. HRMS Analysis

High-resolution mass spectrometry was performed on an Agilent 1290 system, equipped with a 6545 Q-TOF mass spectrometer (Wilmington, DE, USA) and a PDA UV detector. The source was equipped with a JetStream ESI probe operating at atmospheric pressure.

### 2.4. Melting Points Analysis

Melting points were recorded on a Metler Toledo MP50 Melting Points system (Greifensee, Switzerland), T_initial_ = 40 °C, heating 3 °C per minute until 200 °C with ME-18552 sample tubes.

### 2.5. UV Analysis and Photostability

UV–VIS absorption spectra of diacids in ethanol were recorded using a Cary 60 Agilent Technologies UV–VIS spectrometer (Wilmington, DE, USA) at a concentration of 10 μM. For the photostability study, samples (10 μM, EtOH) were irradiated during one hour into a Rayonet© RPR-200 (λ = 300 nm, P = 8.32 W/m², stirring, T = 35 °C) (SNE Ultraviolet Co., Branford, CT, USA) using 14 RPR-3000A lamps (SNE Ultraviolet Co., Branford, CT, USA; RPR-3000A). Then, UV spectra were recorded and the absorbance loss were calculated in percentage at the λ_max_.

### 2.6. Antiradical Activities

The determination of the radical scavenging activity of the diacids was determined via 2,2-diphenyl-1-picrylhydrazyl (DPPH) assay. These tests involved adding potential antiradical molecule solution in ethanol at different concentrations to homogeneous DPPH solution. The study was performed under stirring for 7 h 25 min on the following concentration scale: 400, 200, 100, 50, 25, and 12.5 µM. Every 5 min, the absorbance was measured at 520 nm. At the end, the percentage curves of %DPPH (blue) and %reduced DPPH (green) were plotted in Regressi^®^ software using an average of the last six points. The amount needed to reduce the initial number of DPPH free radicals by half, i.e., EC_50_, was provided by the crossing point of %DPPH (blue) and %reduced DPPH (green).

### 2.7. Experimental Descriptions

#### 2.7.1. Ferulic Diacid. Yield: 80%

^1^H NMR (300 MHz, 25 °C, (CD_3_)_2_CO): δ (ppm) = 7.62 (1H, s, H-3), 7.35 (1H, d, *J* = 2.0 Hz, H-9), 7.21 (1H, dd, *J* = 2.0 and 8.3 Hz, H-5), 6.89 (1H, d, *J* = 8.3 Hz, H-6), 3.86 (3H, s, H-10).

^13^C NMR (75 MHz, 25 °C, (CD_3_)_2_CO): δ (ppm) = 168.5 and 165.9 (C-1 and C-1′), 150.1 (C-7), 148.3 (C-8), 142.1 (C-3), 125.7 (C-4), 125.5 (C-2), 124.4 (C-5), 116.0 (C-6), 113.6 (C-9), 56.1 (C-10).

Melting point: 190–192 ± 0.1 °C.

TOF MS ES+: [M + H]^+^ for C_11_H_11_O_6_: *m/z* 239.0556; found: *m/z* 239.0555.

#### 2.7.2. *p*-Coumaric Diacid. Yield: 71%

^1^H NMR (300 MHz, 25 °C, (CD_3_)_2_CO): δ (ppm) = 7.62 (1H, s, H-3), 7.58 (2H, d, *J* = 8.6 Hz, H-5 and H-5′), 6.91 (2H, d, *J* = 8.7 Hz, H-6 and H-6′).

^13^C NMR (75 MHz, 25 °C, (CD_3_)_2_CO): δ (ppm) = 168.5 and 165.9 (C-1 and C-1′), 160.8 (C-7), 141.7 (C-3), 132.9 (C-5 and C-5′), 125.4 (C-4), 124.4 (C-2), 116.7 (C-6 and C-6′).

Melting point: 183–186 ± 0.1 °C.

TOF MS ES+: [M + H]^+^ for C_10_H_9_O_5_: *m/z* 209.0450; found: *m/z* 209.0450.

#### 2.7.3. Sinapic Diacid. Yield: 68%

^1^H NMR (300 MHz, 25 °C, (CD_3_)_2_CO): δ (ppm) = 7.99 (1H, s, H-10), 7.61 (1H, s, H-3), 7.06 (2H, s, H-5 and H-5′), 3.84 (6H, s, H-8 and H-8′).

^13^C NMR (75 MHz, 25 °C, (CD_3_)_2_CO): δ (ppm) = 168.9 and 165.8 (C-1 and C-1′), 148.8 (C-6 and C-6′), 142.3 (C-3), 139.8 (C-7), 124.9 (C-4), 124.5 (C-2), 108.7 (C-5 and C-5′), 56.1 (C-8 and C-8′).

Melting point: 172–175 ± 0.1 °C.

TOF MS ES+: [M + H]^+^ for C_12_H_13_O_7_: *m/z* 269.0661; found: *m/z* 269.0661.

#### 2.7.4. Caffeic Diacid. Yield: 60%

^1^H NMR (300 MHz, 25 °C, (CD_3_)_2_CO): δ (ppm) = 7.54 (1H, s, H-3), 7.24 (1H, d, *J* = 2.2 Hz, H-9), 7.08 (1H, dd, *J* = 2.2 and 8.3 Hz, H-5), 6.89 (1H, d, *J* = 8.3 Hz, H-6).

^13^C NMR (75 MHz, 25 °C, (CD_3_)_2_CO): δ (ppm) = 168.4 and 165.9 (C-1 and C-1′), 149.0 (C-7), 146.1 (C-8), 141.8 (C-3), 125.9 (C-4), 124.7 (C-2), 124.4 (C-5), 116.9 (C-9), 116.3 (C-6).

Melting point: 162–164 ± 0.1 °C.

TOF MS ES+: [M + H]^+^ for C_10_H_9_O_6_: *m/z* 225.0399; found: *m/z* 225.0399.

## 3. Results and Discussion

**Figure 2:**
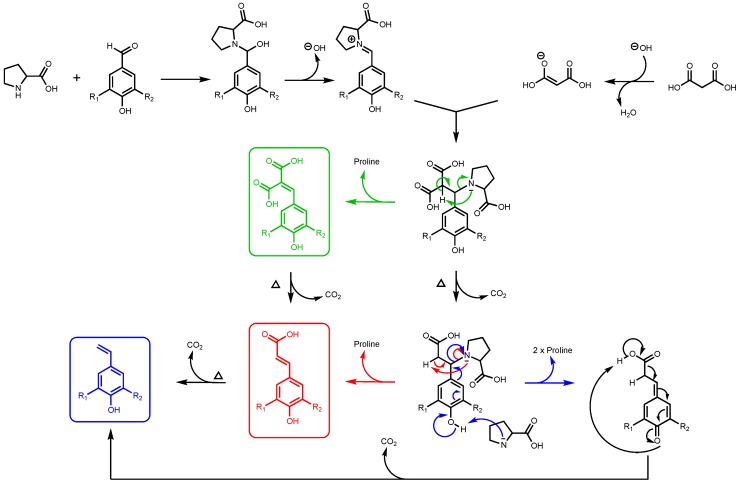
Formation of *p*-hydroxycinnamic diacids, *p*-hydroxycinnamic acids, and the corresponding styrene derivatives through Knoevenagel–Doebner condensation using malonic acid, *p*-hydroxybenzaldehydes, and proline as catalyst (R_1_: H, OMe; R_2_: H, OH, OMe).

It is important to note that, for this study, ferulic diacid production from vanillin and malonic acid was selected as the model reaction. As reported in a previous work by Peyrot et al., *p*-hydroxycinnamic acids can be obtained through Knoevenagel–Doebner condensation between *p*-hydroxybenzaldehydes and malonic acid in ethanol using proline as catalyst [27]. It was observed that the mechanism of the *p*-hydroxycinnamic diacid formation strongly depends on the reaction conditions as depicted in Figure 2.

**Figure 3:**
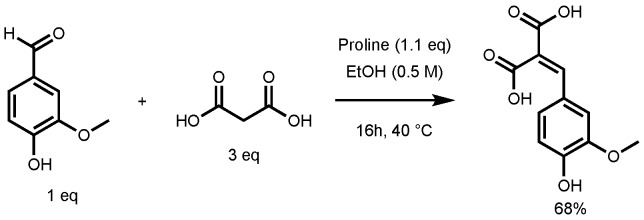
Non-optimized reaction conditions to access ferulic diacid.

In their study, the formation of ferulic diacid in 68% yield was observed using the following conditions: 1 equivalent (eq) of vanillin in ethanol (0.5 M), 3 eq of malonic acid, and 1.1 eq of proline, 16 h at 40 °C (Figure 3) [27]. This result served as foundation for the optimization of the *p*-hydroxycinnamic diacid synthesis.

The Knoevenagel–Doebner condensation of malonic acid with *p*-hydroxybenzaldehydes is known to be highly dependent on four parameters: temperature (Temp), reaction time (t), Malonic acid equivalents (MEq), and proline equivalents (PEq) [27]. In order to determine the impact of these four variables on the conversion to ferulic diacid, a design of experiment (DoE) based on a D-optimal design consisting of 28 experiments, including a triplicate at the central point to evaluate the reproducibility, was performed. Parameters and their variations are reported in Table 1.

The relationship between these variables and the response was given by a second-order polynomial equation (Equation (1)) where Y represents the conversion to ferulic diacid, a_0_ is a constant, x_i_ and xj are the variables and a_i_, a_ii_, and a_ij_ are the linear, quadratic, and interaction coefficients, respectively. After computational treatment to fit the raw results to the second-order polynomial equation, variance (ANOVA) was used for validation of the model with the analysis of R², Q², and lack of fit (LOF) test. R² indicates how well the model fits with the experimental data, Q² gives an estimate on the precision for future predictions and LOF shows whether the model error can be compared to the replicates errors.

(1) $$Y=a_{0}+\underset{i}{\sum }a_{i\;}x_{i}+\underset{i}{\sum }a_{ii\;}x_{i}{}^{2}+\underset{ij}{\sum }a_{ij\;}x_{i}x_{j}$$

**Equation (1).** Second-order polynomial equation for the D-Optimal design.

**Scheme 1:**
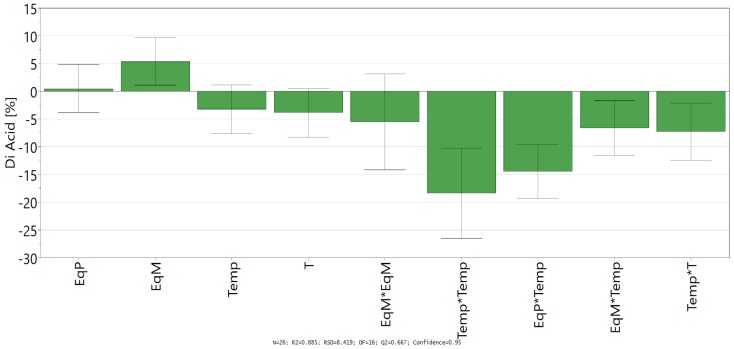
Regression coefficient of quadratic model.

D-Optimal design led to a good fit and prediction of the model with a coefficient of determination R² = 0.885 (>0.6) and a coefficient of cross-validation Q² = 0.667 (>0.5). The lack of fit (*p* > 0.05) shows the low replicate errors of the model. Finally, analysis of variance (ANOVA) shows an acceptable correlation between the response (conversion into ferulic diacid) and the variables with a *p*-value below 0.05, which confirm the statistical significance of the polynomial regression. Coefficients of the models (a_i_, a_ii_, and a_ij_), given in Scheme 1, allowed the determination of the influence of the linear parameter, their square terms, and their quadratic effects.

Looking at the independent variables, only the equivalent of malonic acid shows a significant positive impact on the conversion of vanillin into ferulic diacid, while the time and temperature have a negative one. Likewise, all the significant square terms and quadratic effects with significance exhibit negative impact in the conversion into ferulic diacid, especially all the interactions comprising time and/or temperature. Those results confirm the negative influence of time and temperature on the conversion of vanillin into ferulic diacid as these two factors favor the decarboxylation of the latter into ferulic acid. Equation of the model (Equation (2)) was determined by integrating the coefficients into Equation (1):

(2) $$\begin{array}{c} Y=81.05+0.50\left(PEq\right)+5.39\left(MEq\right)-3.25\left(Temp\right)-3.88\left(t\right)-5.50{\left(MEq\right)}^{2}\\ -18.38{\left(Temp\right)}^{2}-14.49\left(PEq*Temp\right)-6.66\left(MEq-Temp\right)-7.28\left(Temp*t\right) \end{array}$$

**Equation 2.** Equation of the model.

**Scheme 2:**
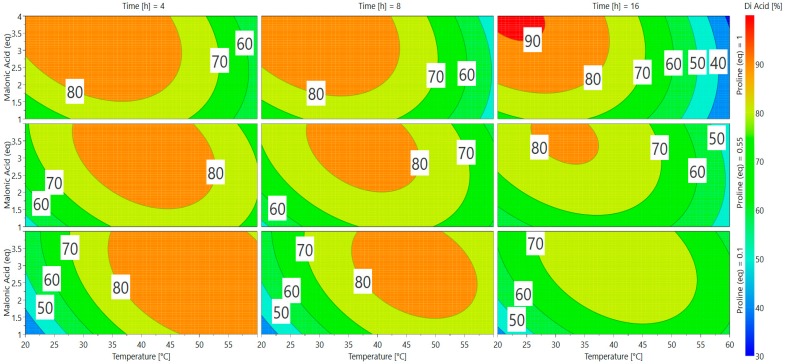
Response surfaces for the conversion of vanillin into ferulic diacid.

A direct visualization of the results from Equation (2) can be obtained via response surface methodology (RSM) in Scheme 2. Optimal responses are represented by red and orange areas and many parameters can be used to achieve appealing conversion (>75%). The software gave two predictions as the best conditions for vanillin conversion into ferulic diacid (Table 2).

In order to choose between the two possible optimal sets of conditions, we selected the one that required the minimum equivalent of reagents while limiting waste to dispose of. Applying these two green chemistry principles, the use of only 1 eq of malonic acid seemed to be the most appropriate choice. Furthermore, the quantity of proline having no significant effect on the conversion, use of 0.1 eq was preferred. Finally, the best conditions appeared to be: 60 °C, 4 h, 1 equivalent of malonic acid, and 0.1 equivalent of proline (Table 2, entry 2), which led to a conversion into ferulic diacid of 80%, very close to the 81% predicted by the software (determined by ^1^H NMR; Table 3, Entry 2). This model has been validated in triplicate and the diacid conversion was repeatable (ESI Table S1).

According to the optimal conditions obtained through the DoE, ferulic diacid conversion, calculated by ^1^H NMR, was 80%. As the crude mixture was basically made up of unreacted vanillin and ferulic diacid (and traces of ferulic acid, 1%), the purification step was greatly simplified. After purification on a C18-reversed phase silica gel column, using H_2_O/MeOH eluant instead of classic organic solvents, the isolated yield of ferulic diacid was 80%. In a recycling perspective, vanillin was also recovered at the end of the purification step and could be reused in another reaction, thus significantly limiting waste, in accordance with green chemistry principles.

This method allowed to access ferulic diacid with enhanced atom economy compared to Peyrot et al. conditions as not only the equivalents of malonic acid were reduced three-fold, but also the proline amount was divided 11 times, turning proline from a reagent to a catalyst [27]. Moreover, the heating time was reduced four-fold, thus reducing the energetic demand of our synthetic procedure. To determine more precisely the improvement brought by the optimized procedure in terms of atom economy, the process mass intensity (PMI) has been calculated (Table 4; details in Table S3 in the ESI) [38]. In this calculation, according to its definition, all of the matter, except water, involved in the reaction—such as reagents, solvents, treatment solution—is considered. The sum of these masses necessary to produce 10 mmol of ferulic diacid is divided by the mass of 10 mmol of ferulic diacid (i.e., 2.382 g). The lower the PMI, the higher the atom economy. As the solvent of the reaction (i.e., EtOH), could be eventually recovered and recycled, the PMI calculation has been carried out considering both the reagents and the solvent (Table 4, entry 1), and the reagents only (Table 4, entry 2). For the two conditions, crude diacids were purified with the same procedure (C18-reversed phase silica gel column, H_2_O/MeOH), so these steps were not considered in PMI calculations.

Data in Table 4 show that the optimized synthetic procedure gets the best PMI scores (9.7 vs. 13.4 and 1.4 vs. 3.6). Moreover, if one considers the recyclability of the reaction solvent, the atom economy is even higher.

**Figure 4:**
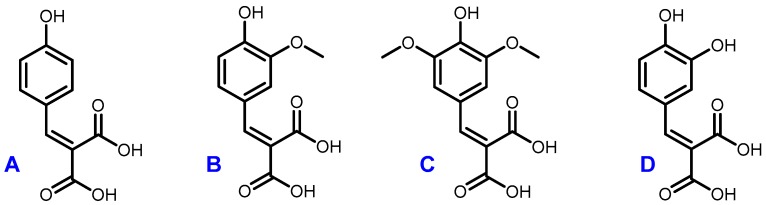
Structures of coumaric diacid (**A**), ferulic diacid (**B**), sinapic diacid (**C**) and caffeic diacid (**D**).

The optimal conditions (60 °C, 4 h, 1 equivalent of malonic acid, and 0.1 equivalent of proline) were then applied to the other *p*-hydroxybenzaldehydes (Figure 4). They provided the corresponding *p*-hydroxycinnamic diacids in good yields (60%–80%) while limiting the presence of undesirable byproducts (Table 5).

**Figure 5:**
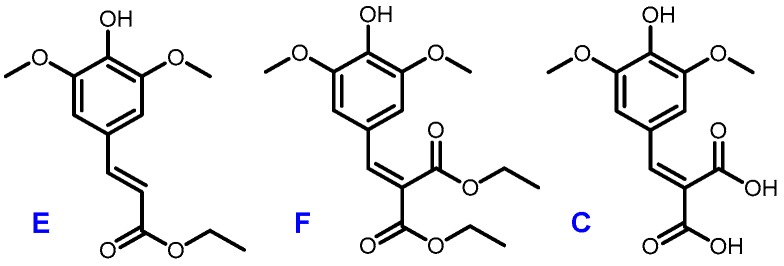
Structures of ethyl sinapate (**E**), diethyl sinapate (DES, **F**) and sinapic diacid (**C**).

A recent work by Horbury et al. focused on two sinapic acid derivatives: ethyl sinapate (ES) and diethyl sinapate (DES) (Figure 5) [10]. These bio-based molecules were highlighted as an exceptional promise for nature-inspired UV filters in next generation sunscreen formulations. As these two possess a high structural similarity with sinapic diacid, we explored the UV potential of the *p*-hydroxycinnamic diacids previously obtained and benchmarked them against DES.

**Figure 6:**
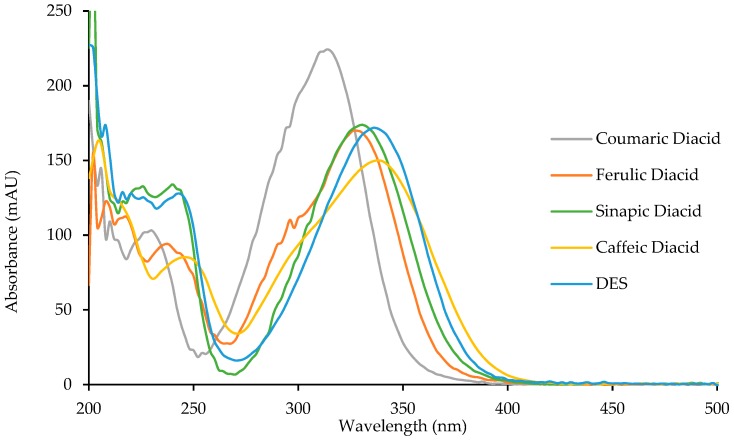
UV spectra of *p*-hydroxycinnamic diacids and DES.

To study the potential anti-UV properties of *p*-hydroxycinnamic diacids, UV analysis were carried out in ethanol (10^−5^ M). Spectra of the four diacids are reported in Figure 6 and their λ_max_ were measured and are reported in Table 6.

Diacids exhibit interesting properties as UV filters for both the UVB region (280–315 nm) and the UVA region (315–400 nm). Particularly, caffeic diacid has a very interesting wavelength coverage from 270 to 400 nm, making it the most promising UV filter among the diacids synthesized herein. Moreover, coumaric diacid, whose coverage is narrower than that of caffeic diacid, showed the higher absorbance intensity of the series. As described by Horbury et al. for DES and ES, photostability is an important parameter when describing UV filters [10]. Indeed, an efficient UV filter must not lose its absorbance at λ _max_ upon UV exposure. To assess photostability, one usually performs the UV analysis of a given UV filter in solution after having been exposed to irradiation at 300 nm (P = 8.32 W/m²) for one hour (t_60_) at T = 35 °C. This t_60_ UV spectrum is then compared to the original one (t_0_), and the loss in absorbance is calculated at the λ_max_. Photostability was investigated for the four diacids, DES, and Octinoxate™, a widely used UV filter in sunscreen and employed as an anti-UV-B reference (Table 6). Interestingly, diacids showed a low loss in absorbance (<10%, Table 6), coumaric and caffeic diacids possessing the best photostability with 3.0% and 4.9% of absorbance loss, respectively. Although DES exhibits a λ_max_ of 335 nm and only lose 3.3% of its absorbance upon exposure to irradiation [10] its use as UV filter may be quite limited because of its low water solubility due to the two hydrophobic ester functions. Such water solubility issue is overcome in the case of diacids whose carboxylic acid moieties warrants great water solubility. As for Octinoxate™, not only is its photostability the worst (26.5% loss in absorbance) but it is considered a hazard for coral reefs and is banned from sale and distribution in some Pacific islands, such as Hawaii, by January 1^st^, 2021 [39,40]. To summarize, these results demonstrate the high potential of the diacids as alternative to Octinoxate™.

**Figure 7:**
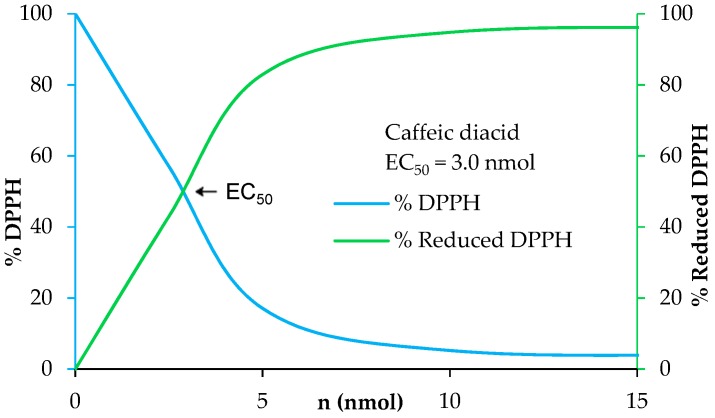
Determination of the antiradical activity of caffeic diacid.

UV filters in sunscreens are doped with antioxidants to prevent and enhance their photo-protection as solar irradiation generates free radicals and damages UV filters leading to a loss of efficiency [41]. Moreover, antioxidants allow skin protection against reactive oxygen species (ROS) induced by UV-B [42]. A benefit for new UV filters would be to have both a good photostability and good antioxidant properties. In this way, we investigated the antioxidant capacities of the *p*-hydroxycinnamic diacids as polyphenols are widely recognized as good antioxidant agents thanks to the ability of phenols to quench free radicals [43]. Antiradical activities were measured through DPPH analysis [44]. These analyses consist in the addition of potential antiradical *p*-hydroxycinnamic diacid solution in ethanol at different concentrations to homogeneous DPPH solution. The amount needed to reduce the initial number of DPPH free radicals by half (i.e., EC_50_) was determined by the crossing point of the “% DPPH” curve (blue) and the “% reduced DPPH” curve (green) (Figure 7). The lower the EC_50_ value, the higher the antioxidant potential. As an example, the antiradical analysis of caffeic diacid is described into Figure 7.

Results for all *p*-hydroxycinnamic diacids are given in Table 7 and are benchmarked against two commercially available antioxidants: BHA and BHT.

BHA and BHT’s EC_50_ values are 6.5 and 13.1 nmol, respectively. *p*-Coumaric diacid did not express any antiradical activity at studied concentrations. These results were expected as Reano et al. demonstrated that coumarate derivatives were very poor radical scavengers [45].

The EC_50_ of ferulic diacid value was determined as being 20.7 nmol, higher than that of BHA and BHT. Sinapic and caffeic diacids exhibits an EC_50_ of 3.9 and 3.0 nmol, respectively, demonstrating that these compounds are much better than commercial references BHA and BHT. Moreover, sinapic diacid activity was also stronger compared to that of DES (32.7 nmol) [10]. In summary, sinapic and caffeic diacids were shown to be great radical scavenger.

## 4. Conclusions

Herein, we report a new synthetic pathway to bio-based *p*-hydroxycinnamic diacids. Wishing to respect green chemistry principles, such as the use of non-toxic solvent and reactants, atom economy with the use of minimal equivalent of proline and malonic acid, and the use of naturally-occurring *p*-hydroxybenzaldehydes, this pathway was optimized on ferulic diacid through a design of experiments. The optimal conditions were then successfully applied to three others naturally-occurring *p*-hydroxybenzaldehydes, providing the four desired bio-based *p*-hydroxycinnamic diacids in good yields (60–80%). The four diacids showed not only interesting UV properties covering the UV-A and UV-B regions but also great photostability, particularly coumaric, and caffeic diacids. Coumaric diacid showed similar UV properties than Octinoxate™ and could be considered as an interesting alternative to this controversial compound. With regards to antiradical activity, sinapic and caffeic diacids proved to be potent antioxidant and potential alternative to BHA [46] and BHT [47] that are now classified as endocrine disruptors, carcinogens, and are withdrawn from the market in some countries. With regards to their valuable properties, sinapic and caffeic diacids could be seriously considered as potential new antioxidant agents in cosmetic, biomaterial or food industry.

## Figures and Tables

**Table 1:** Parameters and their variation corresponding to defined levels of the DoE.

	Levels		
Variables	–1	0	1
Temperature (°C)	20	40	60
Reaction Time (h)	4	8	16
Equivalent of malonic acid	1	2	4
Equivalent of proline	0.1	0.55	1

**Table 2:** Theorical optimal conditions for vanillin conversion into ferulic diacid according to the DoE software.

Entry	T (°C)	T (h)	Malonic Acid (eq)	Proline (eq)	Conversion (%)
1	25	16	4	1	91
2	60	4	1	0.1	81

**Table 3:** Optimization of the proline-catalyzed Knoevenagel condensation of vanillin and malonic acid in ethanol (0.5 M).

	Original Conditions ^[b]^	Optimized Conditions ^[c]^
Proline (eq)	1.1	0.1
Malonic acid (eq)	3	1
T (°C)	40	60
T (h)	16	4
Remaining vanillin (%)	25	17
Conversion into diacid ^[a]^ (%)	68	80
Conversion into monoacid ^[a]^ (%)	7	1

[a] Conversions were determined from the ^1^H NMR spectrum of the crude reaction mixture. [b] According to Peyrot et al. conditions [27]. [c] Optimal set of conditions obtained through the DoE.

**Table 4:** PMI score for the conversion of vanillin in ferulic diacid.

Entry	Calculation Mode	PMI for Original Conditions ^[a]^	PMI for Optimized Conditions ^[b]^
1	Reagents/solvent	13.4	9.7
2	Reagents	3.6	1.4

[a] According to Peyrot et al. conditions [27]. [b] Optimal set of conditions obtained through the DoE.

**Table 5:** Conversion rate and isolated yields of *p*-hydroxycinnamic diacids.

Substrates	Phenolic Diacids	Conversion (%) ^[a]^	Yield (%) ^[b]^
Vanillin	Ferulic diacid	80	80
Syringaldehyde	Sinapic diacid	70	68
4-Hydroxybenzaldehyde	*p*-Coumaric diacid	72	71
3,4-Dihydroxybenzaldehyde	Caffeic diacid	61	60

[a] Conversions were determined from the ^1^H NMR spectrum of the crude reaction mixture. [b] Yields were calculated from isolated product after purification.

**Table 6:** UV analysis: λ max and photostability.

	λ Max (nm) ^[a]^	Absorbance	Loss in Absorbance at λ Max (%) ^[a]^
Ferulic Diacid	328	170	1.9^[b]^
Coumaric Diacid	314	224	3.0^[b]^
Sinapic Diacid	330	174	8.9^[b]^
Caffeic Diacid	340	150	4.9^[b]^
Octinoxate	310	240	26.5^[c]^
DES [10]	336	172	3.3

[a] Performed in ethanol (10^–5^ M). [b] ESI Figure S3. [c] ESI Figure S4.

**Table 7:** Antiradical activities.

	EC_50_ (nmol)
Ferulic diacid	20.7
Sinapic diacid	3.9
*p*-Coumaric diacid	ND ^[a]^
Caffeic diacid	3.0
BHA	6.5
BHT	13.1

[a] No EC_50_ was obtained at studied concentrations.

## Supplementary Materials

The following are available online at https://www.mdpi.com/2076-3921/9/4/331/s1; Figure S1. Structures and numeration of coumaric diacid (**A**), ferulic diacid (**B**), sinapic diacid (**C**), and caffeic diacid (**D**); Figure S2. UV spectra of diacids; Figure S3. Photostability of diacids; Figure S4. Photostability of Octinoxate; Figure S5. Determination of the antiradical activity of diacids; Figure S6. ^1^H spectrum of aromatic zone for ferulic diacid conversion; Figure S7. ^1^H spectrum of ferulic diacid; Figure S8. ^13^C spectrum of ferulic diacid; Figure S9. ^1^H spectrum of coumaric diacid; Figure S10. ^13^C spectrum of coumaric diacid; Figure S11. ^1^H spectrum of sinapic diacid; Figure S12. ^13^C spectrum of sinapic diacid; Figure S13. ^1^H spectrum of caffeic diacid; Figure S14. ^13^C spectrum of caffeic diacid; Figure S15. HRMS spectrum of ferulic diacid; Figure S16. HRMS spectrum of coumaric diacid; Figure S17. HRMS spectrum of sinapic diacid; Figure S18. HRMS spectrum of caffeic diacid, Table S1. DOE experiments; Table S2. Application of the optimal conditions designed by the DOE to the *p*-hydroxybenzaldehydes; Table S3. PMI score calculation between our procedure (Entry 33, Table S1) and Peyrot et al. [27] conditions (Entry 34, Table S1).
