# Supplementary material for: Sustainable Synthesis of p-Hydroxycinnamic Diacids through Proline-Mediated Knoevenagel Condensation in Ethanol: An Access to Potent Phenolic UV Filters and Radical Scavengers
Source: Antioxidants (Basel). 2020 Apr 18;9(4):331. doi: 10.3390/antiox9040331 (PMC7222392; doi:10.3390/antiox9040331)
Supplement: Supplementary file 1 [file antioxidants-09-00331-s001.pdf]

Sustainable synthesis of *p*-hydroxycinnamic diacids through proline-mediated Knoevenagel condensation in ethanol: an access to potent phenolic UV filters and radical scavengers.

Benjamin RIOUX[1], Cédric PEYROT[1], Matthieu M. MENTION[1], Fanny BRUNISSEN[1], Florent ALLAIS*[1]

[1] URD Agro-Biotechnologies Industrielles, CEBB, AgroParisTech, 51110, Pomacle, France.

* Correspondence: florent.allais@agroparistech.fr; Tel: +33 (0) 3 52 62 04 62

Total number of Pages: 17 (Pages S1-S17)
Total number of Figures: 18 (Figures S1-S18).
Total number of Tables: 2 (Tables S1-S2).

Table of contents

General ... 3

Figure S1. Structures and numeration of coumaric diacid (A), ferulic diacid (B), sinapic diacid (C) and caffeic diacid (D). ... 3

Ferulic diacid ... 4

Coumaric diacid ... 4

Sinapic diacid ... 4

Caffeic diacid ... 4

Table S1. DOE experiments. ... 5

Table S2. Application of the optimal conditions designed by the DOE to the *p*-hydroxybenzaldehydes. ... 6

Table S3. PMI score calculation between our procedure (Entry 33, Table S1) and Peyrot *et al.*[12] conditions (Entry 34, Table S1). ... 6

Figure S2: UV spectra of diacids. ... 7

Figure S3: Photostability of diacids. ... 8

Figure S4: Photostability of Octinoxate. ... 9

Figure S5: Determination of the antiradical activity of diacids. ... 10

NMR analysis ... 11

Figure S6: ^{1}H spectrum of aromatic zone for ferulic diacid conversion ... 11

Figure S7: ^{1}H spectrum of ferulic diacid ... 12

Figure S8: ^{13}C spectrum of ferulic diacid ... 12

Figure S9: ^{1}H spectrum of coumaric diacid ... 13

Figure S10: ^{13}C spectrum of coumaric diacid ... 13

Figure S11: ^{1}H spectrum of sinapic diacid ... 14

Figure S12: ^{13}C spectrum of sinapic diacid 14
Figure S13: ^{1}H spectrum of caffeic diacid 15
Figure S14: ^{13}C spectrum of caffeic diacid 15
HRMS analysis 16
Figure S15: HRMS spectrum of ferulic diacid 16
Figure S16: HRMS spectrum of coumaric diacid 16
Figure S17: HRMS spectrum of sinapic diacid 17
Figure S18: HRMS spectrum of caffeic diacid 17

General

All reagents were purchased from Sigma–Aldrich, TCI, Merck or VWR and used as received. Solvents were purchased from Thermo Fisher Scientific and VWR. Deuterated dimethylsulfoxide (DMSO-*d6* <0.02%H_2O) was purchased from Euriso-top. Evaporations were conducted under reduced pressure at temperature below 40 °C. Column chromatography was carried out with an automated flash chromatography (PuriFlash 4100, Interchim) and pre-packed INTERCHIM PF-30C18HP-HP-F0080 (30 µm silica gel) columns. UV/visible absorption spectra of diacids in ethanol were recorded using a UV/visible spectrometer Cary 60 Agilent Technologies at a concentration of 10 µM. For the photostability study, samples (10 µM, EtOH) were irradiated during one hour into a Rayonet© RPR-200 (λ = 300 nm, P = 8.32 W/m², stirring, T = 35 °C) using 14 RPR-3000A lamps (SNE Ultraviolet Co Branford Ct USA RPR-3000A). Then, UV spectra were recorded and the absorbance loss were calculated in percentage at the λ_{max}. NMR analyses were recorded on a Bruker Fourier 300. ^{1}H NMR spectra of samples were recorded in DMSO (residual peak at δ = 2.50 ppm) or $(CD_3)_2CO$ (residual signal at δ = 2.05 ppm) at 300 MHz, chemical shifts were reported in parts per million relatives to the solvent residual peak. ^{13}C NMR spectra of samples were recorded at 75 MHz in DMSO (residual signal at δ = 39.52 ppm) or $(CD_3)_2CO$ (residual peak at δ = 206.26 and 29.84 ppm). ^{1}H NMR crude calculation conversion was recorded by integration of diacids' aromatic signal. Melting points were recorded on a Metler Toledo MP50 Melting Points system, $T_{initial}$ = 40 °C, heating 3 °C / minute until 200 °C with ME-18552 sample tubes. Mass spectrometry analysis were recorded on an Agilent Technologies 6545 Q-TOF LC/MS. The determination of the diacids' antiradical activities were determined *via* 2,2-diphenyl-1-picrylhydrazyl (DPPH) assay. These tests involve adding potential antiradical molecule solution in ethanol at different concentration to homogeneous DPPH solution. The amount needed to reduce the initial number of DPPH free radicals by half, i.e. EC_{50}, is provided by the crossing point of % DPPH (blue) and % reduced DPPH (green)

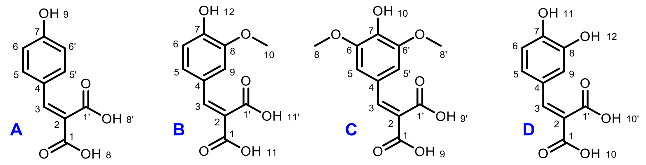

Figure S1. Structures and numeration of coumaric diacid (A), ferulic diacid (B), sinapic diacid (C) and caffeic diacid (D).

Ferulic diacid

Synthesis: Vanillin (225 mg, 1.5 mmol), malonic acid (153.9 mg, 1.5 mmol, 1 eq), proline (17.1 mg, 0.15 mmol, 0.1 eq) and ethanol (3 mL, 0.5 M) were stirred at 60 °C for 4 hours. The reaction mixture was then evaporated under vacuum. Conversion yields were determined by proton NMR. Crude mixture was then purified by column chromatography on C-18 reverse silica gel using appropriate eluent system (H_2O:methanol 80:20). 282 mg of a yellow/white powder was recovered (80%).
^{1}H NMR (300 MHz, 25°C, $(CD_3)_2CO$): δ (ppm) = 7.62 (1H, s, H-3), 7.35 (1H, d, *J* = 2.0 Hz, H-9), 7.21 (1H, dd, *J* = 2.0 and 8.3 Hz, H-5), 6.89 (1H, d, *J* = 8.3 Hz, H-6), 3.86 (3H, s, H-10).
^{13}C NMR (75 MHz, 25°C, $(CD_3)_2CO$): δ (ppm) = 168.5 and 165.9 (C-1 and C-1'), 150.1 (C-7), 148.3 (C-8), 142.1 (C-3), 125.7 (C-4), 125.5 (C-2), 124.4 (C-5), 116.0 (C-6), 113.6 (C-9), 56.1 (C-10).
Melting point: 190-192 ± 0.1 °C.
TOF MS ES+: $[M+H]^+$ for $C_{11}H_{11}O_6$: *m/z* 239.0556; found: *m/z* 239.0555.
UV: λ max = 328 nm.
DPPH: EC_{50} = 20.72 nmol.

Coumaric diacid

Synthesis: 4-hydroxybenzaldehyde (181.4 mg, 1.5 mmol), malonic acid (153.9 mg, 1.5 mmol, 1 eq), proline (17.1 mg, 0.15 mmol, 0.1 eq) and ethanol (3 mL, 0.5 M) were stirred at 60 °C for 4 hours. The reaction mixture was then evaporated under vacuum. Conversion yields were determined by proton NMR. Crude mixture was then purified by column chromatography on C-18 reverse silica gel using appropriate eluent system (H_2O:methanol 80:20). 219.3 mg of a yellow/white powder was recovered (71%).
^{1}H NMR (300 MHz, 25°C, $(CD_3)_2CO$): δ (ppm) = 7.62 (1H, s, H-3), 7.58 (2H, d, *J* = 8.6 Hz, H-5 and H-5'), 6.91 (2H, d, *J* = 8.7 Hz, H-6 and H-6').
^{13}C NMR (75 MHz, 25°C, $(CD_3)_2CO$): δ (ppm) = 168.5 and 165.9 (C-1 and C-1'), 160.8 (C-7), 141.7 (C-3), 132.9 (C-5 and C-5'), 125.4 (C-4), 124.4 (C-2), 116.7 (C-6 and C-6').
Melting point: 183-186 ± 0.1 °C.
TOF MS ES+: $[M+H]^+$ for $C_{10}H_9O_5$: *m/z* 209.0450; found: *m/z* 209.0450.
UV: λ max = 314 nm.
DPPH: EC_{50} = no EC_{50} was obtained at studied concentrations.

Sinapic diacid

Synthesis: Syringaldehyde (270.5 mg, 1.5 mmol), malonic acid (153.9 mg, 1.5 mmol, 1 eq), proline (17.1 mg, 0.15 mmol, 0.1 eq) and ethanol (3 mL, 0.5 M) were stirred at 60 °C for 4 hours. The reaction mixture was then evaporated under vacuum. Conversion yields were determined by proton NMR. Crude mixture was then purified by column chromatography on C-18 reverse silica gel using appropriate eluent system (H_2O:methanol 80:20 to 50:50). 269.5 mg of a yellow/white powder was recovered (68%).
^{1}H NMR (300 MHz, 25°C, $(CD_3)_2CO$): δ (ppm) = 7.99 (1H, s, H-10), 7.61 (1H, s, H-3), 7.06 (2H, s, H-5 and H-5'), 3.84 (6H, s, H-8 and H-8').
^{13}C NMR (75 MHz, 25°C, $(CD_3)_2CO$): δ (ppm) = 168.9 and 165.8 (C-1 and C-1'), 148.8 (C-6 and C-6'), 142.3 (C-3), 139.8 (C-7), 124.9 (C-4), 124.5 (C-2), 108.7 (C-5 and C-5'), 56.1 (C-8 and C-8').
Melting point: 172-175 ± 0.1 °C.
TOF MS ES+: $[M+H]^+$ for $C_{12}H_{13}O_7$: *m/z* 269.0661; found: *m/z* 269.0661.
UV: λ max = 330 nm.
DPPH: EC_{50} = 3.86 nmol.

Caffeic diacid

Synthesis: 3,4-dihydroxybenzaldehyde (270.5 mg, 1.5 mmol), malonic acid (153.9 mg, 1.5 mmol, 1 eq), proline (17.1 mg, 0.15 mmol, 0.1 eq) and ethanol (3 mL, 0.5 M) were stirred at 60 °C for 4 hours. The reaction mixture was then evaporated under vacuum. Conversion yields were determined by proton NMR. Crude mixture was then purified by column chromatography on C-18 reverse silica gel using appropriate eluent system (H_2O:methanol 90:10). 197.7 mg of a yellow/white powder was recovered (60%).
^{1}H NMR (300 MHz, 25°C, $(CD_3)_2CO$): δ (ppm) = 7.54 (1H, s, H-3), 7.24 (1H, d, *J* = 2.2 Hz, H-9), 7.08 (1H, dd, *J* = 2.2 and 8.3 Hz, H-5), 6.89 (1H, d, *J* = 8.3 Hz, H-6).
^{13}C NMR (75 MHz, 25°C, $(CD_3)_2CO$): δ (ppm) = 168.4 and 165.9 (C-1 and C-1'), 149.0 (C-7), 146.1 (C-8), 141.8 (C-3), 125.9 (C-4), 124.7 (C-2), 124.4 (C-5), 116.9 (C-9), 116.3 (C-6).
Melting point: 162-164 ± 0.1 °C.
TOF MS ES+: $[M+H]^+$ for $C_{10}H_9O_6$: *m/z* 225.0399; found: *m/z* 225.0399.
UV: λ max = 340 nm.
DPPH: EC_{50} = 2.96 nmol.

N°	Eq Proline	Eq Malonic Acid	Temperature (°C)	Time (h)	m Proline (mg)	m Malonic Acid (mg)	m Vanillin (mg)	V Ethanol (mL)	Vanillin (%) [a]	Diacid (%) [a]	Acid (%) [a]	Other (%) [a]	Total (%)
1	0.1	1	20	4	5.7	51.3	75	1	59	34	2	5	100
2	0.1	4	20	4	5.7	205.2	75	1	55	40	3	2	100
3	1	4	20	4	56.8	205.2	75	1	6	83	3	8	100
4	0.1	1	60	4	5.7	51.3	75	1	19	78	1	2	100
5	0.1	4	60	4	5.7	205.2	75	1	20	71	1	9	100
6	1	1	33.3	4	56.8	5103	75	1	11	78	2	10	100
7	1	2	60	4	56.8	102.6	75	1	5	63	13	18	100
8	0.7	4	60	4	39.7	205.2	75	1	4	90	3	2	100
9	0.6	1	40	4	31.2	51.3	75	1	16	66	6	12	100
10	1	1	20	8	56.8	51.3	75	1	11	61	14	14	100
11	1	4	46.7	8	56.8	205.2	75	1	3	75	12	10	100
12	0.7	1	60	8	39.7	51.3	75	1	13	57	19	12	100
13	0.4	4	20	8	22.7	205.2	75	1	6	82	0	11	100
14	0.1	2.5	40	8	5.7	128.2	75	1	11	79	0	10	100
15	0.1	1	20	16	5.7	51.3	75	1	39	39	5	17	100
16	0.1	4	20	16	5.7	205.2	75	1	53	40	2	5	100
17	0.1	1	60	16	5.7	51.3	75	1	28	60	2	9	100
18	1	1	60	16	56.8	51.3	75	1	15	30	38	17	100
19	0.1	4	60	16	5.7	205.2	75	1	17	68	4	11	100
20	1	4	60	16	56.8	205.2	75	1	11	20	25	45	100
21	1	4	33.3	16	56.8	205.2	75	1	2	89	2	7	100
22	1	2	20	16	56.8	102.6	75	1	4	80	2	14	100
23	1	3	20	16	56.8	153.9	75	1	4	81	3	13	100
24	0.6	1	40	16	31.2	51.3	75	1	19	60	4	17	100
25	0.6	2.5	60	16	31.2	128.2	75	1	10	41	17	32	100
26	0.6	2.5	40	8	31.2	128.2	75	1	1	91	3	5	100
27	0.6	2.5	40	8	31.2	128.2	75	1	2	83	3	11	100
28	0.6	2.5	40	8	31.2	128.2	75	1	2	83	4	11	100
29	0.1	1	60	4	5.7	51.3	75	1	15	77	1	7	100
30	0.1	1	60	5	5.7	51.3	75	1	20	73	1	6	100
31	0.1	1	60	6	5.7	51.3	75	1	18	72	1	9	100
32	0.1	1	60	4	17.1	153.9	225	3	19	75	1	4	100
33	0.1	1	60	4	17.1	153.9	225	3	17	80	1	2	100
34 [b]	1.1	3	40	16	187.3	461.7	225	3	25	68	7	0	100
35	0.1	1	60	2	17.1	153.9	225	3	41	55	3	1	100
36	0.1	1	60	3	17.1	153.9	225	3	36	60	3	1	100

[a] percentage of conversions were determined from the ^{1}H NMR spectrum of the crude reaction mixture [b]: according to Peyrot *et al.*[12]

Table S1. DOE experiments.

Benzaldehyde	Eq Proline	Eq Malonic	Temperature (°C)	Time (h)	m Proline (mg)	m Malonic Acid (mg)	m Aldehyde (mg)	V Ethanol (mL)	Aldehyde (%) [a]	Diacid (%) [a]	Acid (%) [a]	Other (%) [a	Total (%)	Isolated Yield (%)
**Vanilline**	0.1	1	60	4	17.1	153.9	225.0	3	17	80	1	2	100	80
**4-OH**	0.1	1	60	4	17.1	153.9	181.4	3	25	71	3	1	100	71
**3,4-diOH**	0.1	1	60	4	17.1	153.9	205.1	3	30	61	3	6	100	60
**Syringaldehyde**	0.1	1	60	4	17.1	153.9	270.5	3	16	70	12	2	100	68

[a] percentage of conversions were determined from the ^{1}H NMR spectrum of the crude reaction mixture

Table S2. Application of the optimal conditions designed by the DOE to the *p*-hydroxybenzaldehydes.

**Entry 33**				
	n (mol)	Yields (%)	M ($g.mol^{-1}$)	m (g)
Ferulic diacid	1.00E-02	80.06	238.195	2.382
	reactants			
	eq	n (mol)	M ($g.mol^{-1}$)	m (g)
Vanilin	1	1.249E-02	152.149	1.900
Proline	0.1	1.249E-03	115.1305	0.144
Malonic acid	1	1.249E-02	104.0615	1.300
Ethanol	C ($mol.L^{-1}$)	v (mL)	ρ ($g.mL^{-1}$)	m (g)
	0.5	25	0.789	19.711
	$m_{reactants}$ (g)	$m_{ferulic\ diacid}$ (g)	PMI score	
	23.055	2.382	9.7	
Without ethanol:				
	$m_{reactants}$ (g)	$m_{ferulic\ diacid}$ (g)	PMI score	
	3.344	2.382	1.4	

**Entry 34 (Peyrot *et al.*[12])**				
	n (mol)	Yields (%)	M ($g.mol^{-1}$)	m (g)
Ferulic diacid	1.00E-02	67.99	238.195	2.382
	reactants			
	eq	n (mol)	M ($g.mol^{-1}$)	m (g)
Vanilin	1	1.471E-02	152.149	2.238
Proline	1.1	1.618E-02	115.1305	1.863
Malonic acid	3	4.412E-02	104.0615	4.591
Ethanol	C ($mol.L^{-1}$)	v (mL)	ρ ($g.mL^{-1}$)	m (g)
	0.5	29	0.789	23.209
	$m_{reactants}$ (g)	$m_{ferulic\ diacid}$ (g)	PMI score	
	31.900	2.382	13.4	
Without ethanol:				
	$m_{reactants}$ (g)	$m_{ferulic\ diacid}$ (g)	PMI score	
	8.692	2.382	3.6	

Table S3. PMI score calculation between our procedure (Entry 33, Table S1) and Peyrot *et al.*[12] conditions (Entry 34, Table S1).

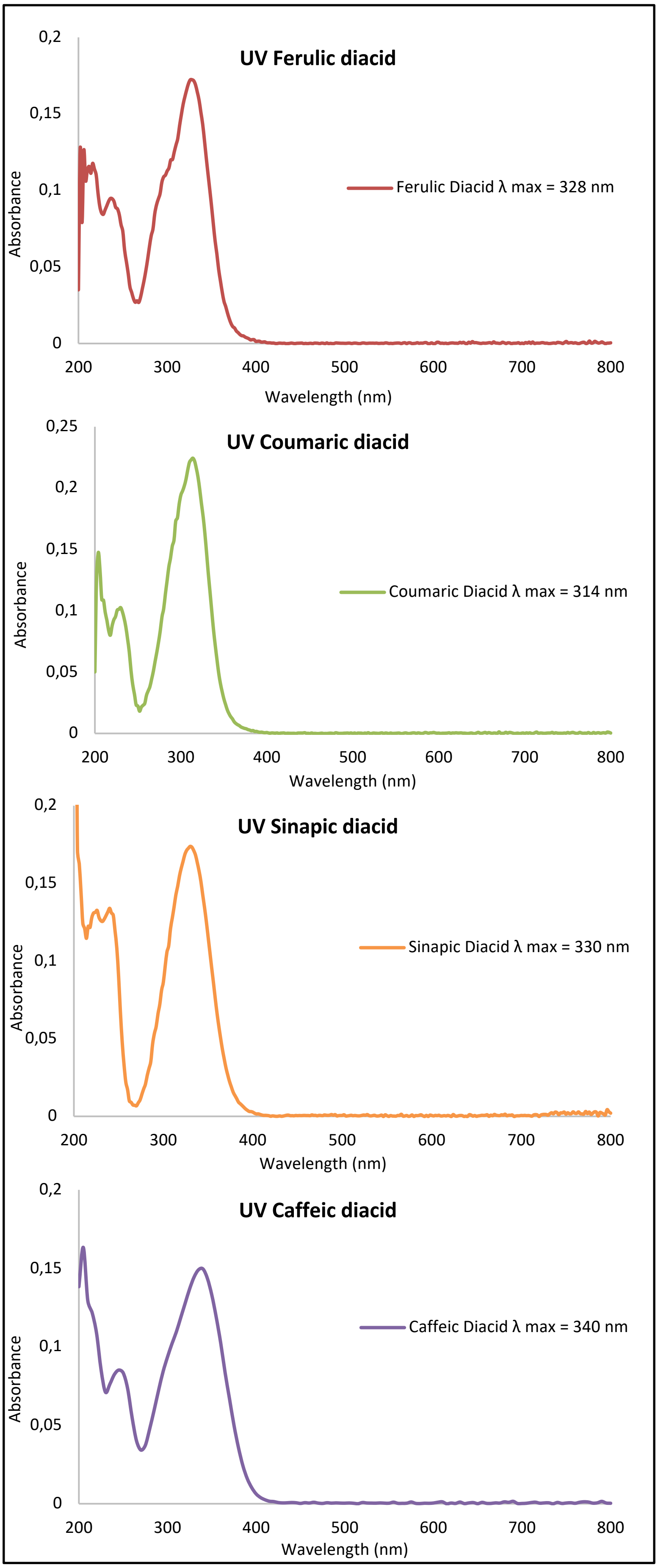

Figure S2: UV spectra of diacids.

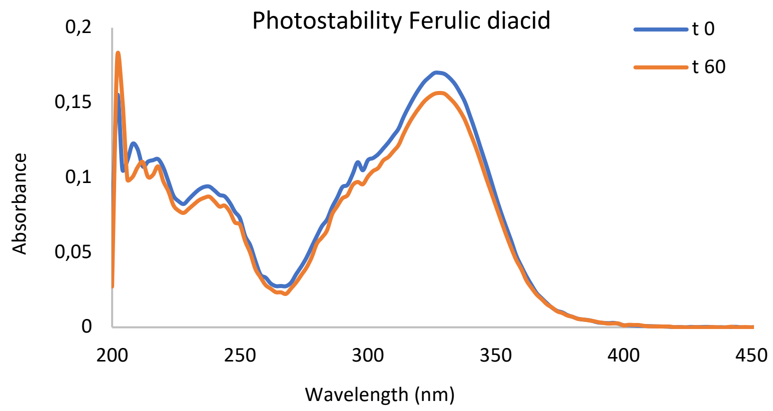

	t 0	t 60
λ max (nm)	328	328
A max	0.170	0.156
Absorbance loss (%):		**7.9**

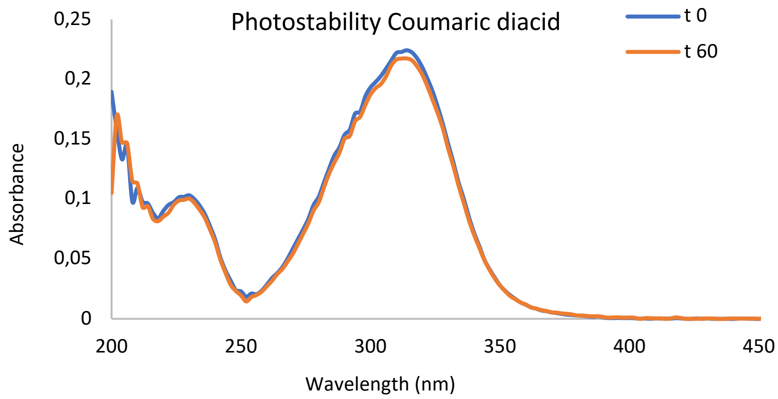

	t 0	t 60
λ max (nm)	314	314
A max	0.224	0.217
Absorbance loss (%):		**3.0**

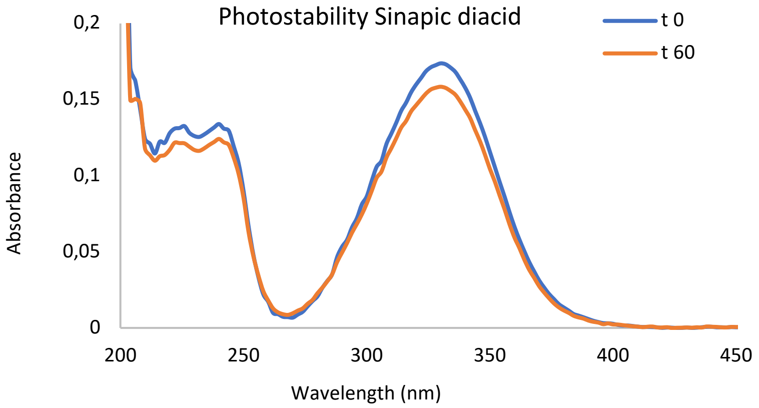

	t 0	t 60
λ max (nm)	330	330
A max	0.174	0.158
Absorbance loss (%):		**8.9**

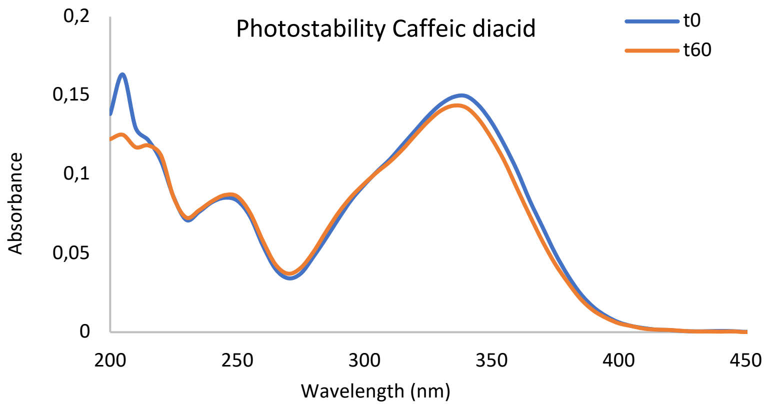

	t 0	t 60
λ max (nm)	340	340
A max	0.150	0.142
Absorbance loss (%):		**4.9**

Figure S3: Photostability of diacids.

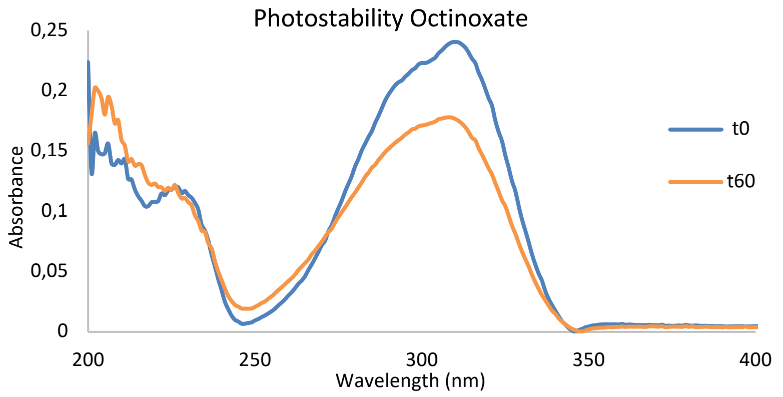

	t 0	t 60
λ max (nm)	310	310
A max	0.240	0.177
Absorbance loss (%):		**26.5**

Figure S4: Photostability of Octinoxate.

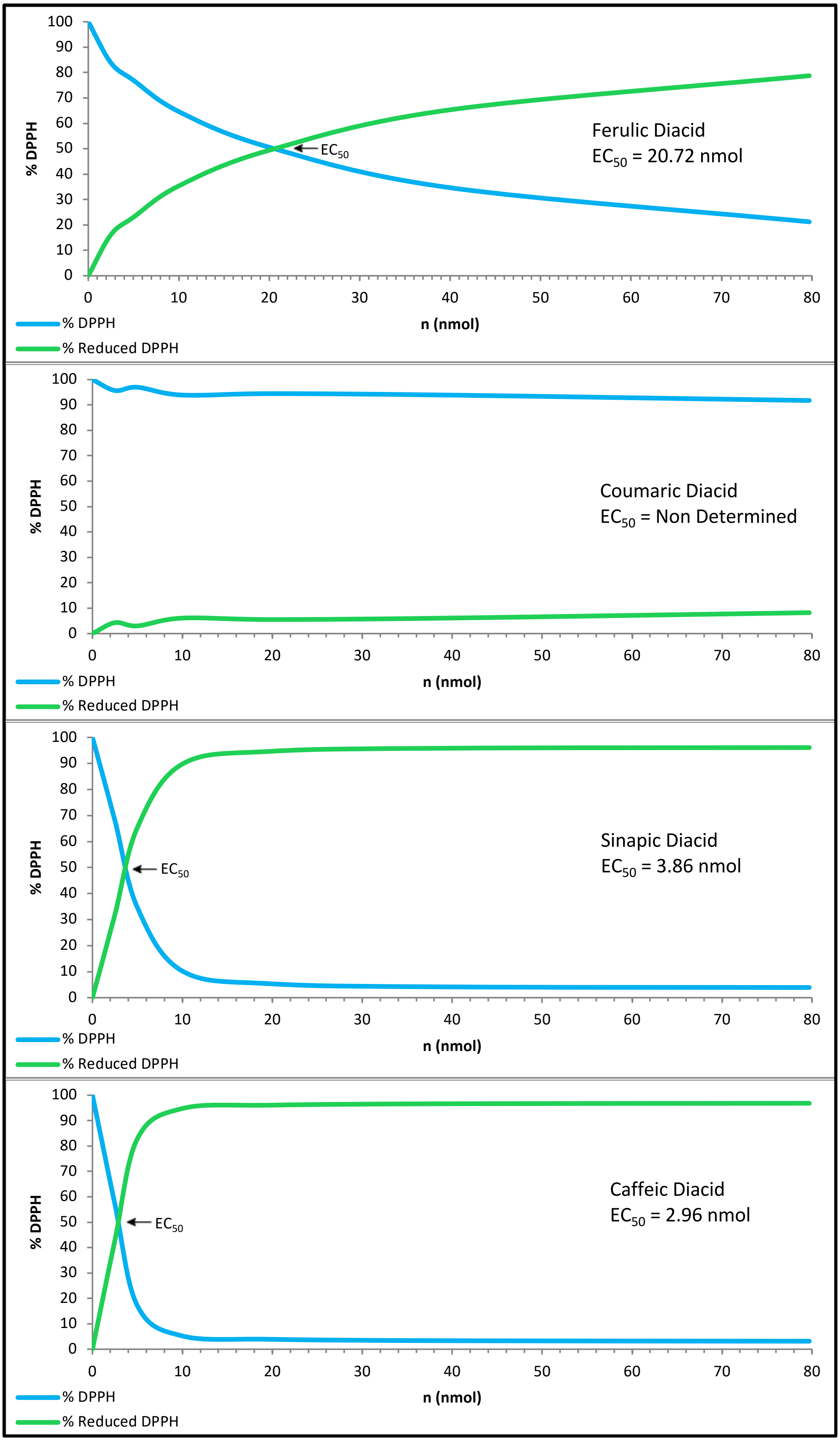

Figure S5: Determination of the antiradical activity of diacids.

NMR analysis

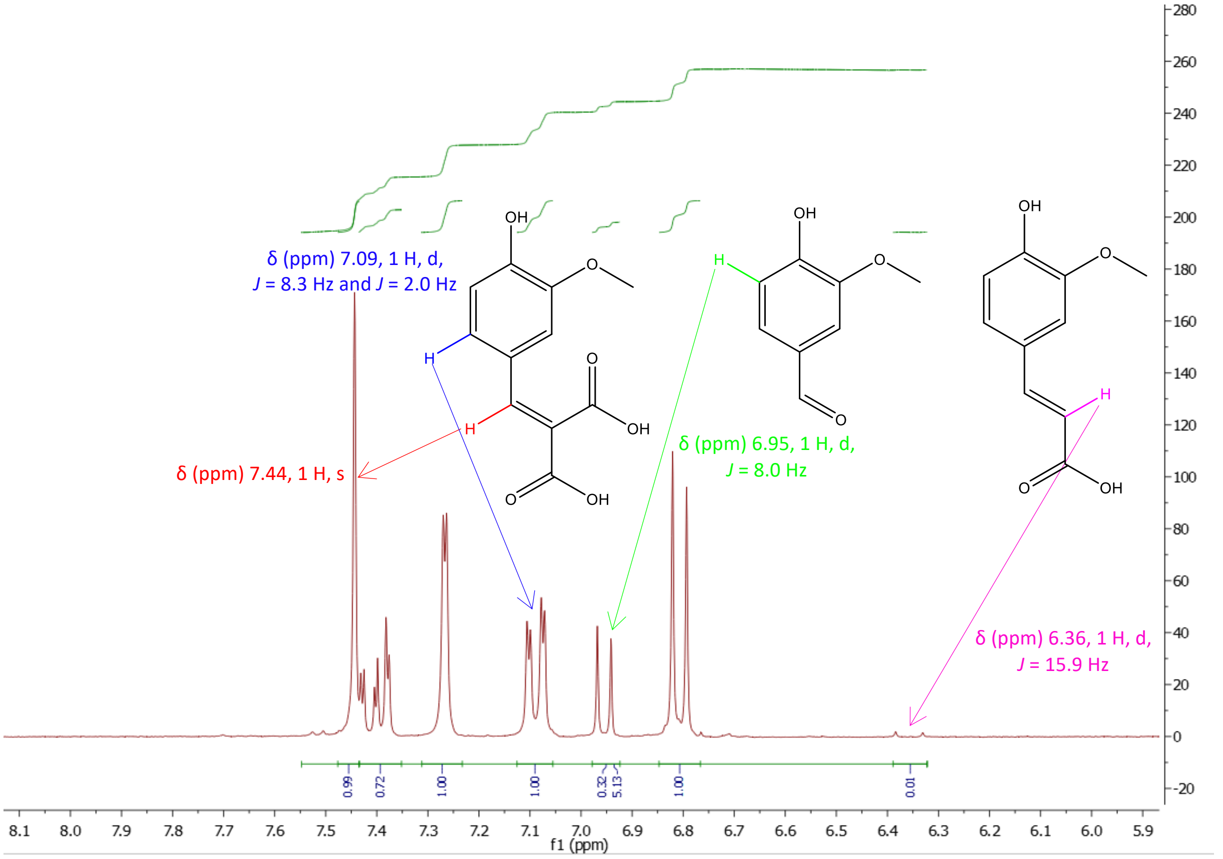

Figure S6: ^{1}H spectrum of aromatic zone for ferulic diacid conversion

Total diacid aromatic protons: vanillin 3, diacid 4, monoacid 5.

Total spectra aromatic protons = 5.13

Vanillin still presents: (0.32x3)/5.13 = 19%

Ferulic diacid conversion: (1x4)/5.13 = 78%

Ferulic acid conversion: (0.01x5)/5.13 = 1%

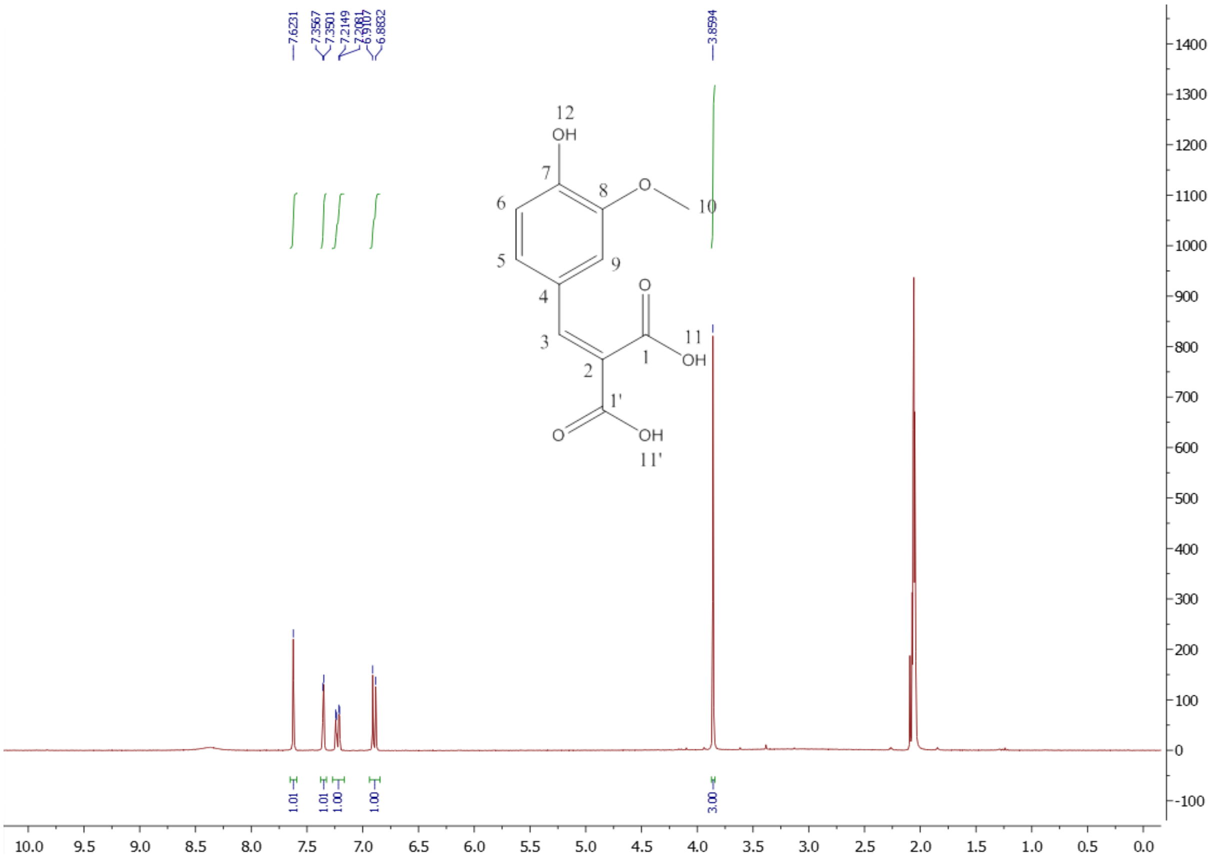

Figure S7: ^{1}H spectrum of ferulic diacid

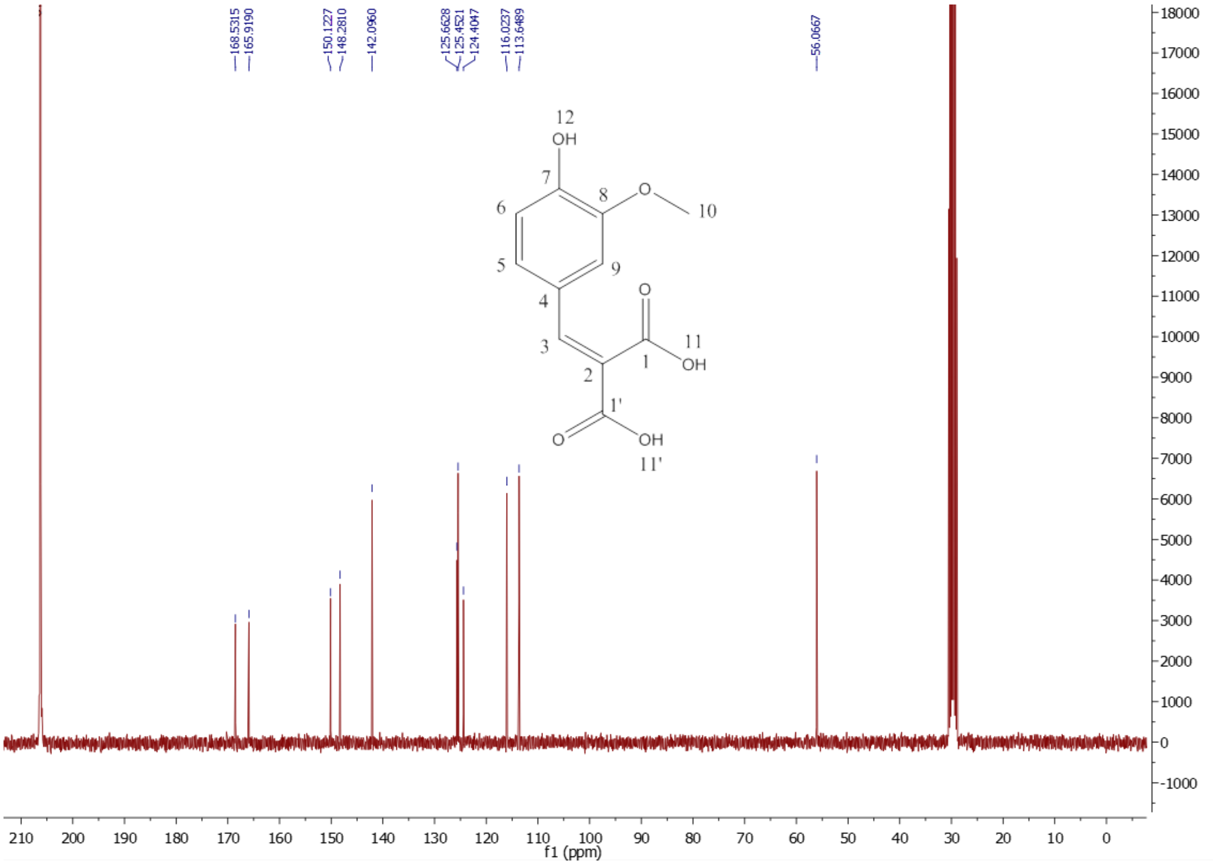

Figure S8: ^{13}C spectrum of ferulic diacid

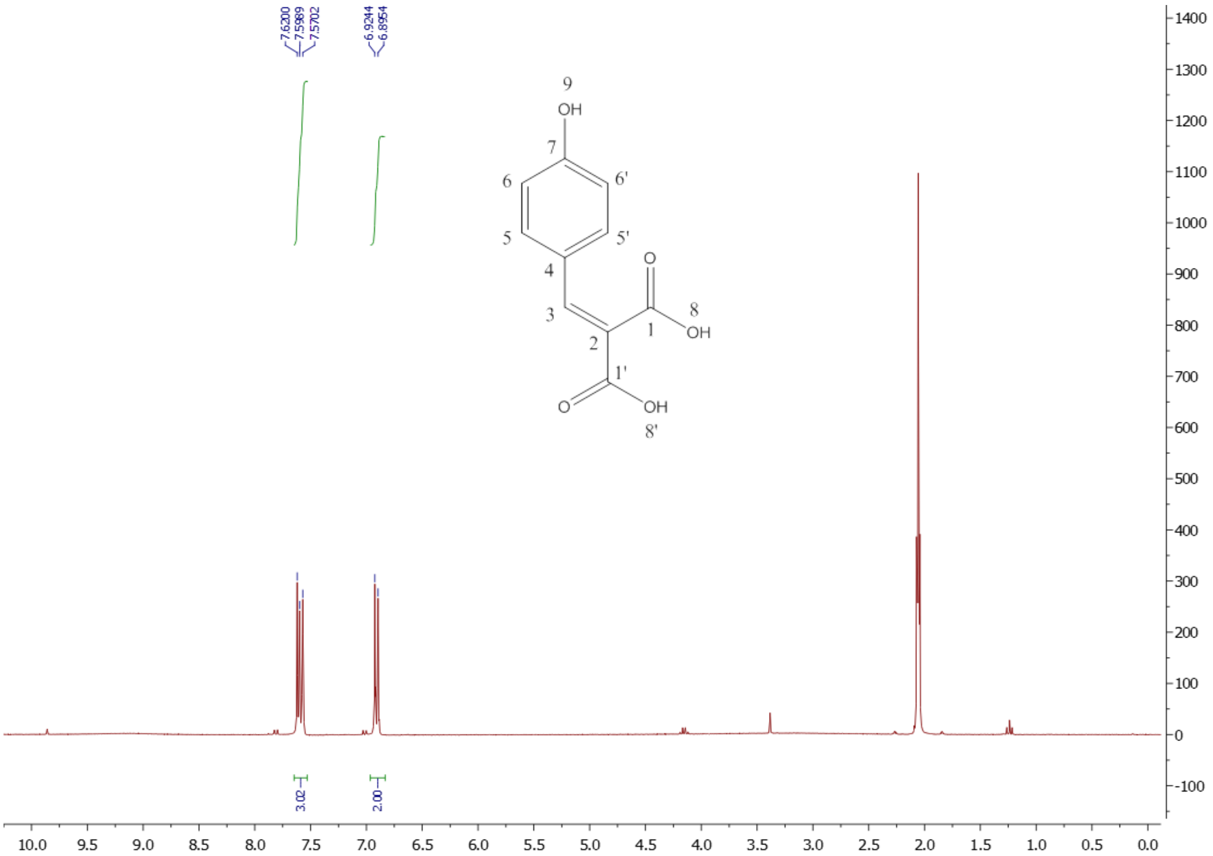

Figure S9: ^{1}H spectrum of coumaric diacid

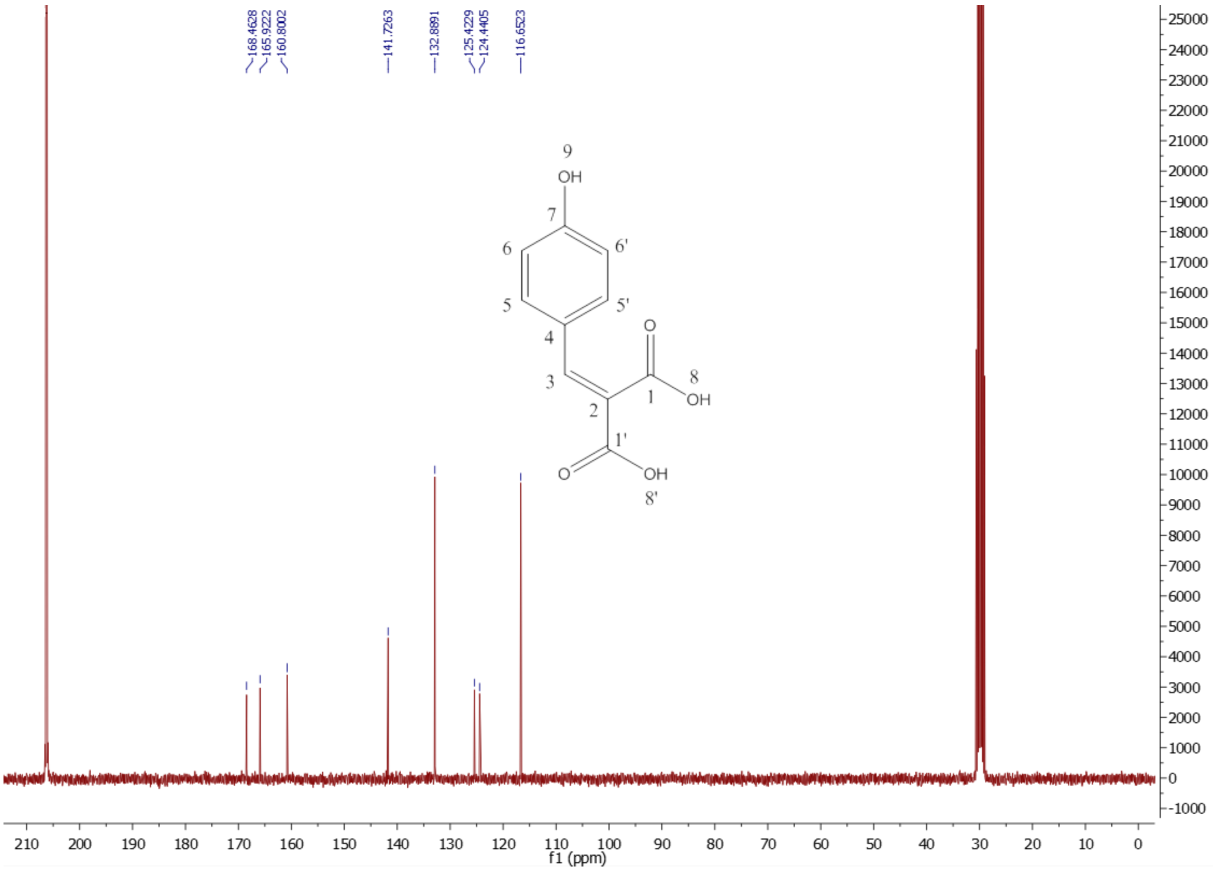

Figure S10: ^{13}C spectrum of coumaric diacid

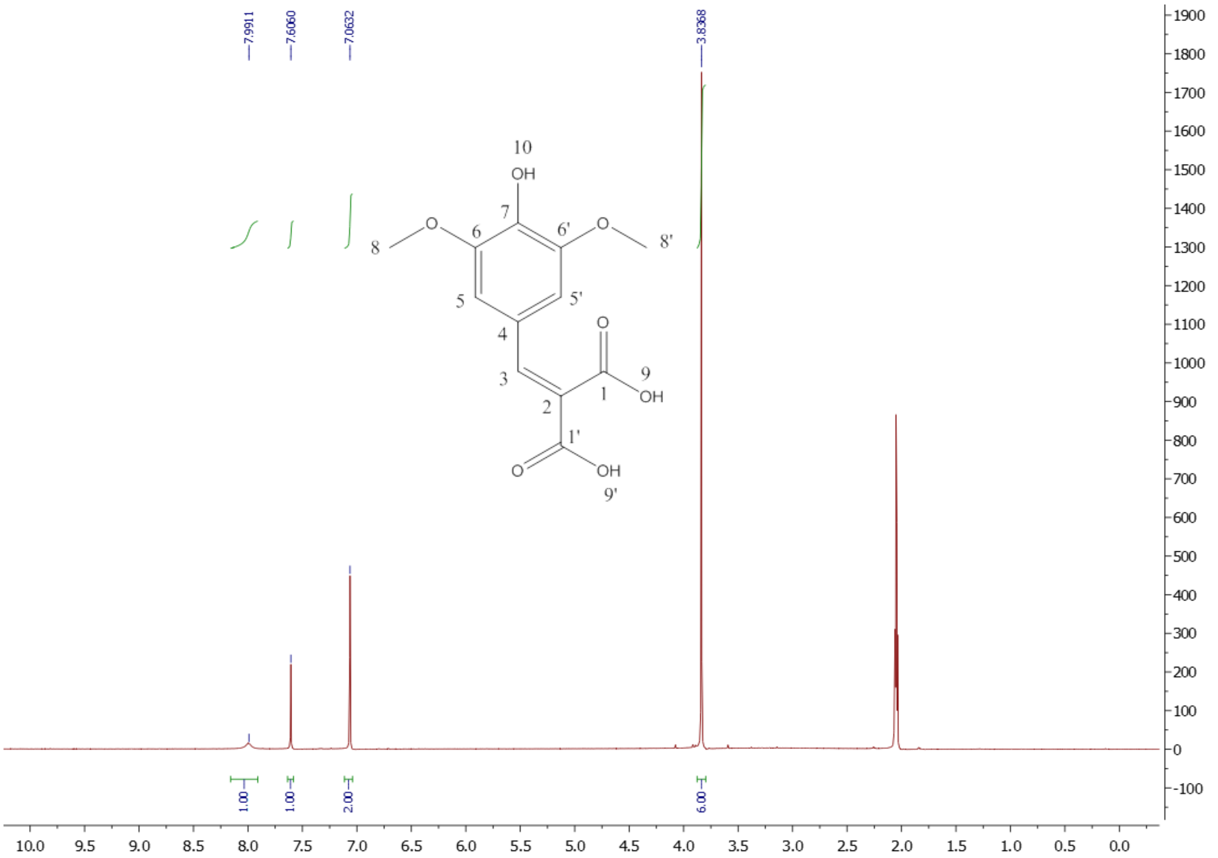

Figure S11: ^{1}H spectrum of sinapic diacid

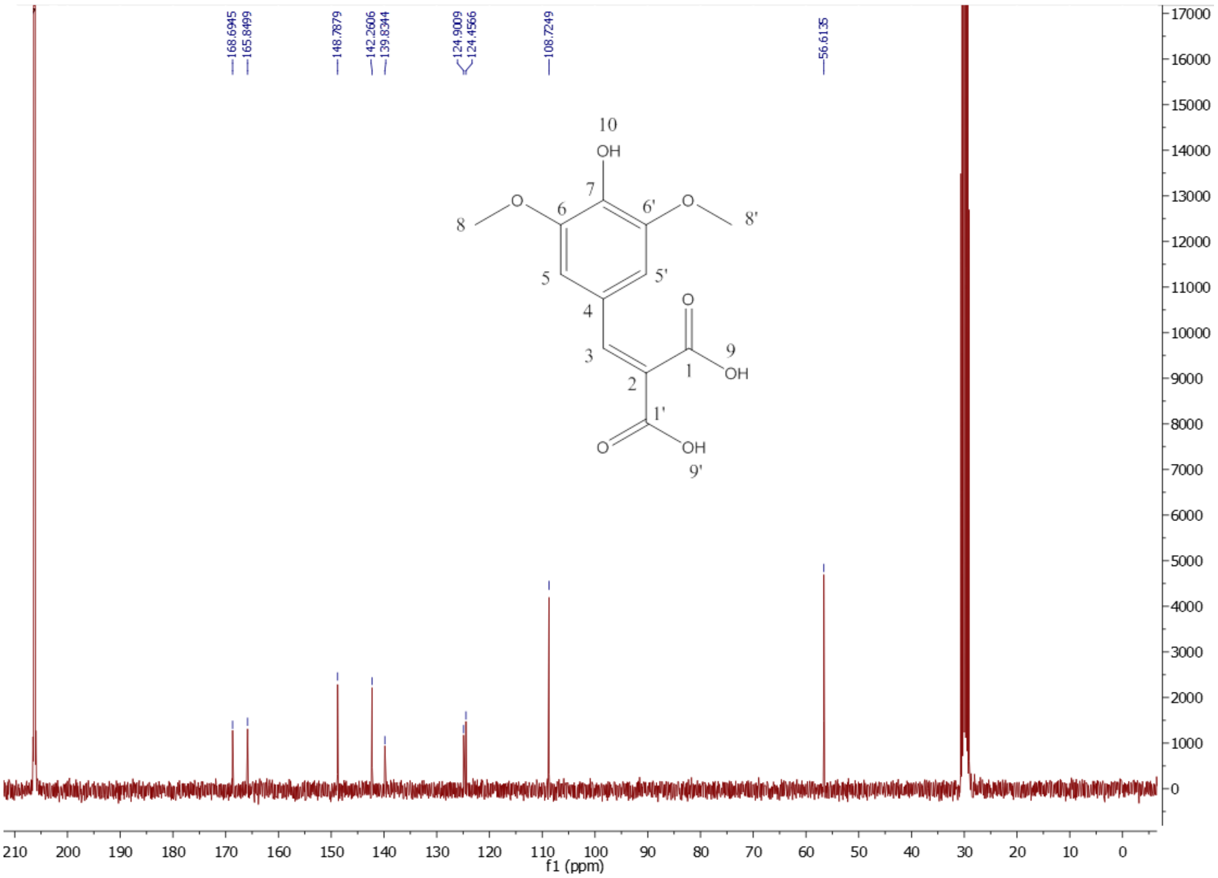

Figure S12: ^{13}C spectrum of sinapic diacid

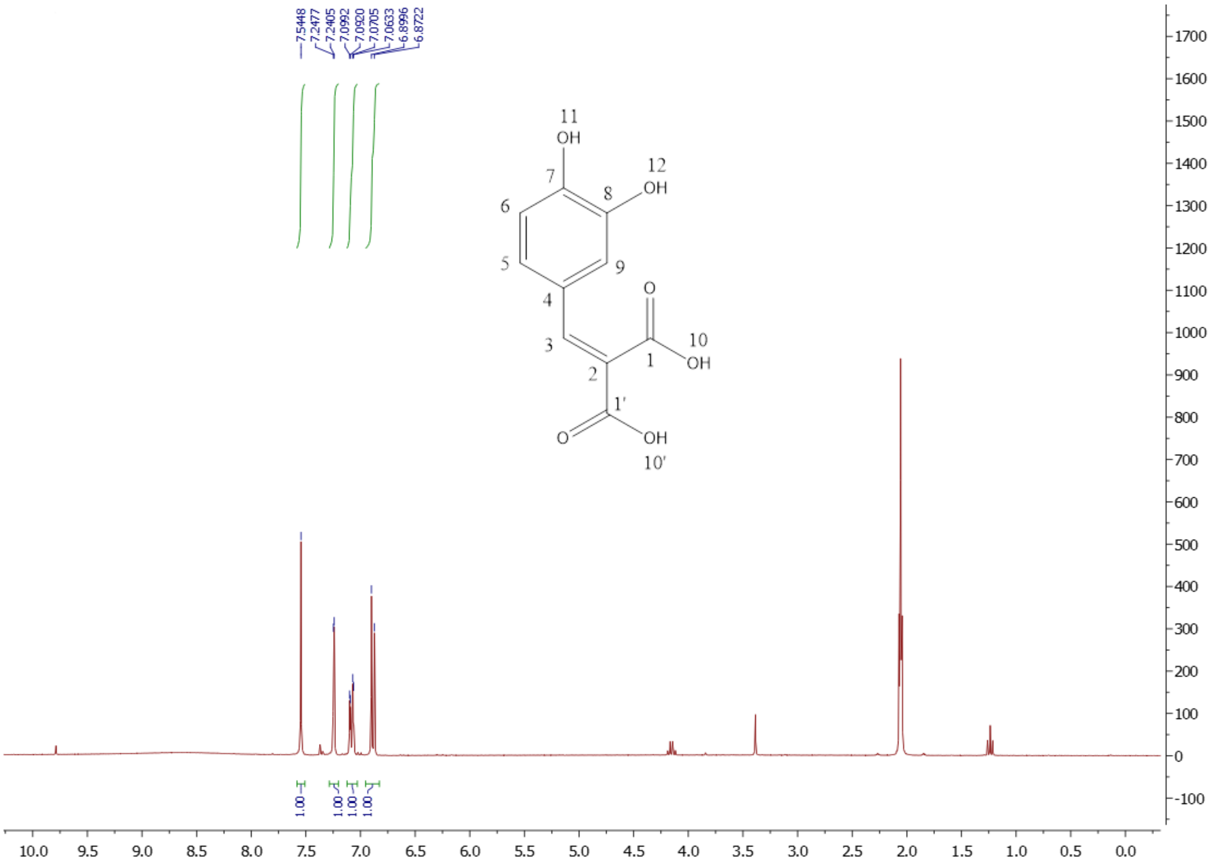

Figure S13: ^{1}H spectrum of caffeic diacid

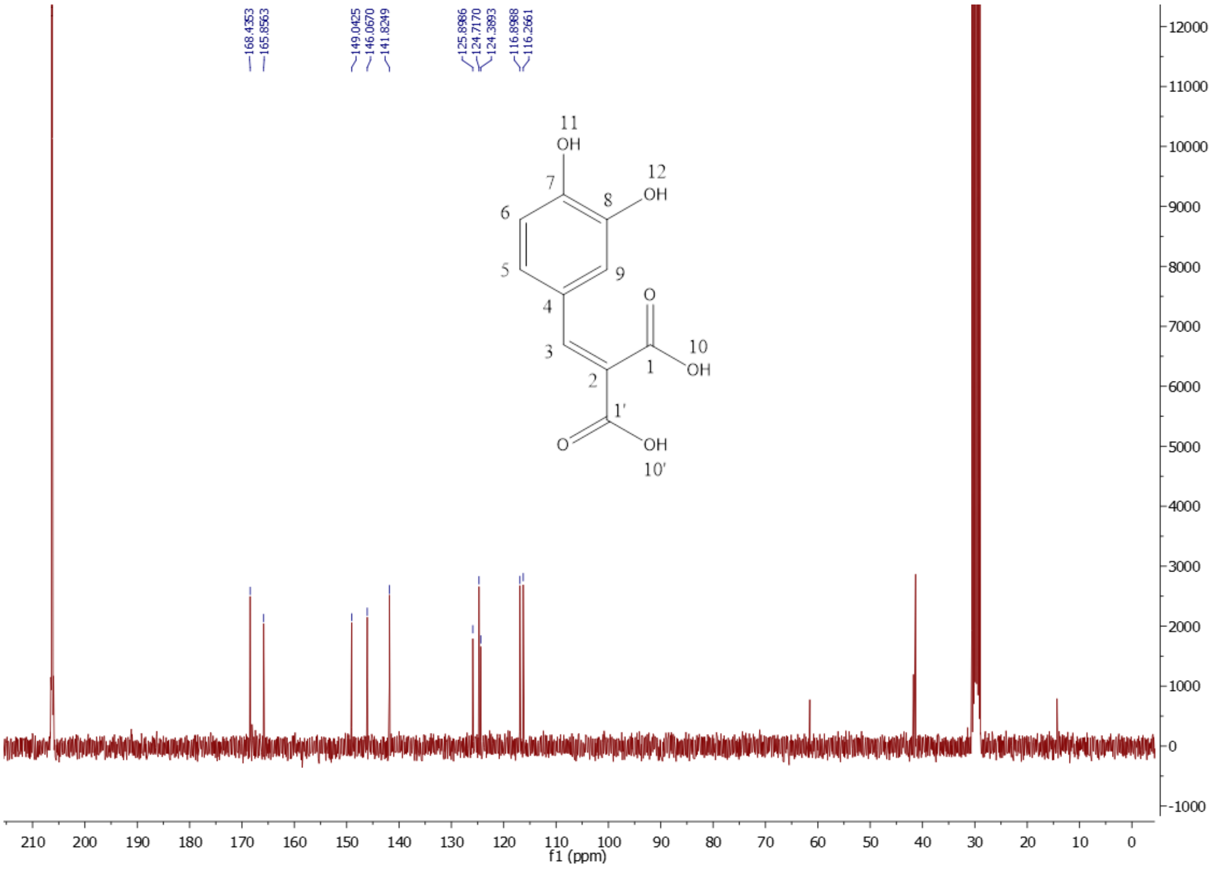

Figure S14: ^{13}C spectrum of caffeic diacid

HRMS analysis

**Sample Name**	RB-ferulic diacid	**Position**	P1-E1	**Instrument Name**	QTOF
**User Name**		**Inj Vol**	1	**InjPosition**	
**Sample Type**	Sample	**IRM Calibration Status**	Success	**Data Filename**	RB-ferulic diacid.d
**ACQ Method**	ZorbaxC18-2019-12-04-H2Oac-ACN-avec-ref.	**Comment**		**Acquired Time**	12/5/2019 4:26:09 PM (UTC+01:00)

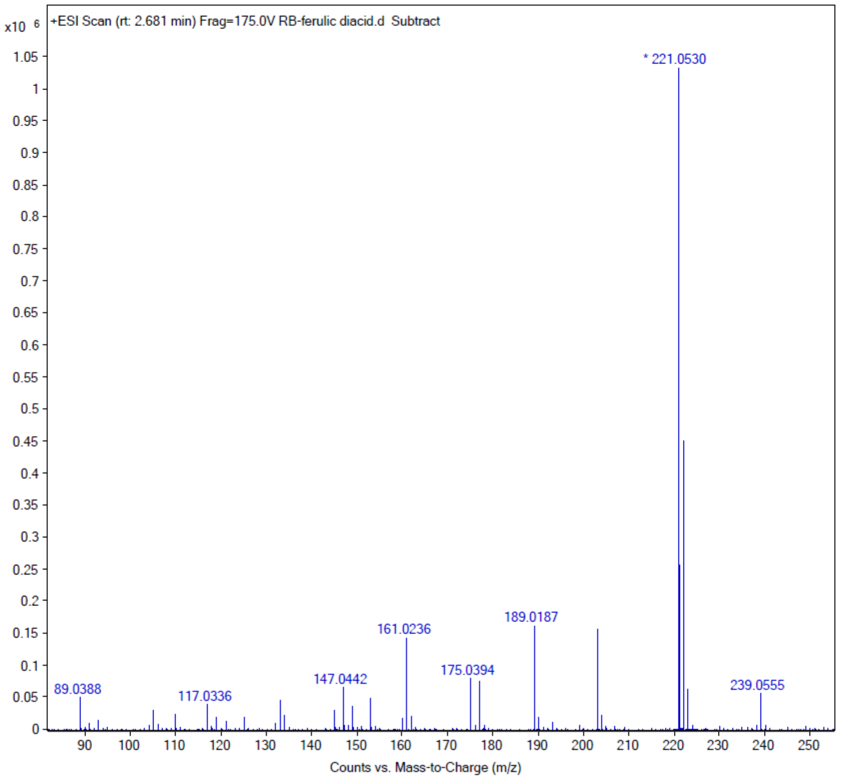

Figure S15: HRMS spectrum of ferulic diacid

**Sample Name**	RB-coumaric diacid	**Position**	P1-E2	**Instrument Name**	QTOF
**User Name**		**Inj Vol**	1	**InjPosition**	
**Sample Type**	Sample	**IRM Calibration Status**	Success	**Data Filename**	RB-coumaric diacid.d
**ACQ Method**	ZorbaxC18-2019-12-04-H2Oac-ACN-avec-ref.	**Comment**		**Acquired Time**	12/5/2019 4:48:14 PM (UTC+01:00)

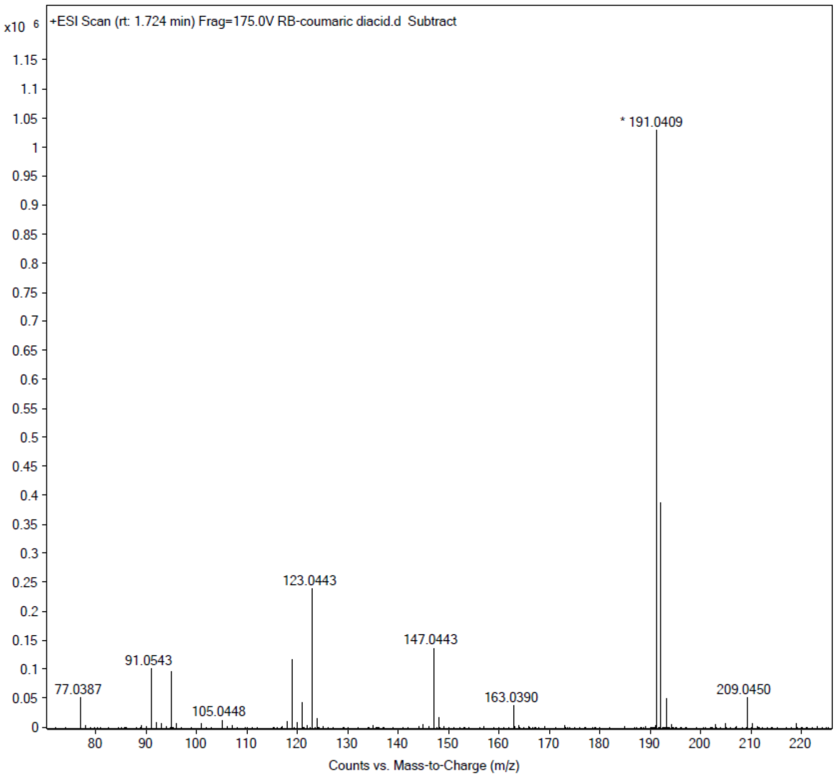

Figure S16: HRMS spectrum of coumaric diacid

**Sample Name**	RB-sinapic diacid	**Position**	P1-E3	**Instrument Name**	QTOF
**User Name**		**Inj Vol**	1	**InjPosition**	
**Sample Type**	Sample	**IRM Calibration Status**	Success	**Data Filename**	RB-sinapic diacid.d
**ACQ Method**	ZorbaxC18-2019-12-04-H2Oac-ACN-avec-ref.	**Comment**		**Acquired Time**	12/5/2019 5:10:17 PM (UTC+01:00)

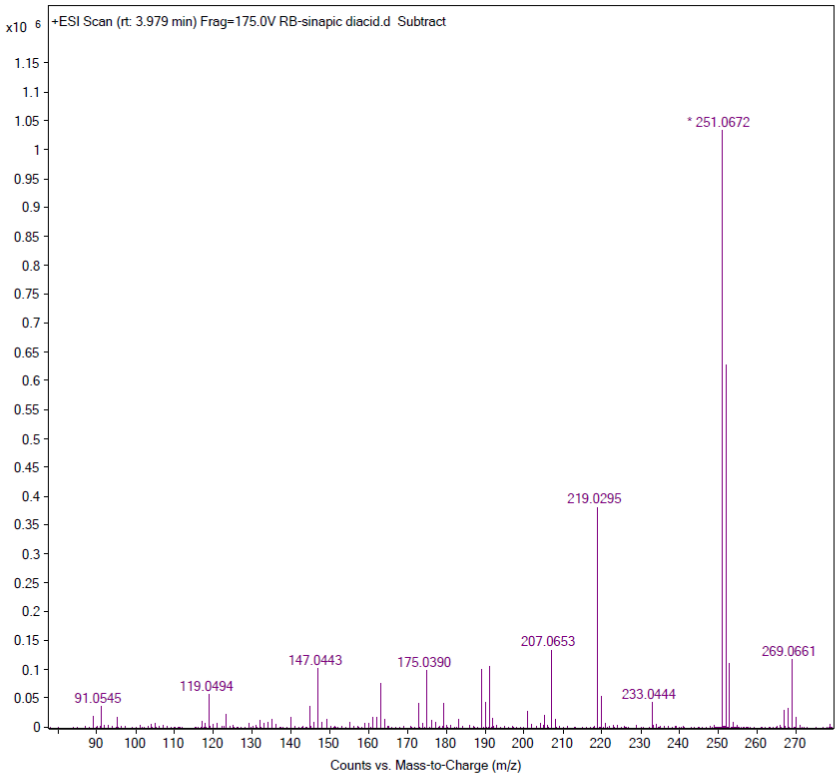

Figure S17: HRMS spectrum of sinapic diacid

**Sample Name**	RB-caffeic diacid	**Position**	P1-E4	**Instrument Name**	QTOF
**User Name**		**Inj Vol**	1	**InjPosition**	
**Sample Type**	Sample	**IRM Calibration Status**	Success	**Data Filename**	RB-caffeic diacid.d
**ACQ Method**	ZorbaxC18-2019-12-04-H2Oac-ACN-avec-ref.	**Comment**		**Acquired Time**	12/5/2019 5:32:21 PM (UTC+01:00)

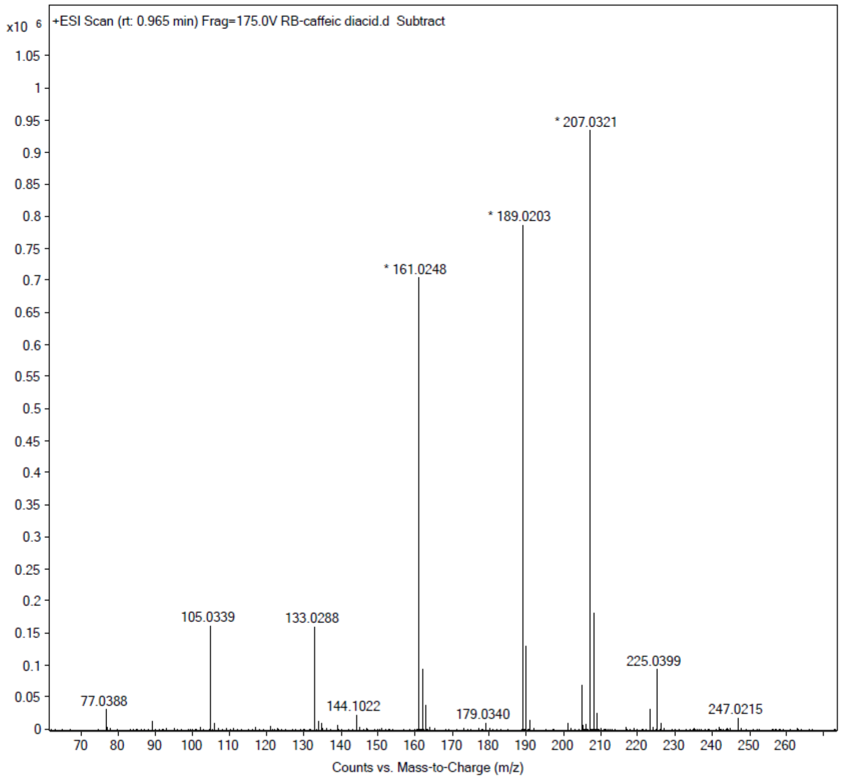

Figure S18: HRMS spectrum of caffeic diacid
